# Wearable Devices in Cardiovascular Care: A Narrative Review of the Transition Toward Predictive, Preventive, Personalized, and Participatory Medicine

**DOI:** 10.3390/healthcare14182894

**Published:** 2026-09-08

**Authors:** Simona Steliana Tudor, Ancuta Elena Tupu, Alice Elena Munteanu, Claudia Simona Stefan, Ionela Daniela Ferțu

**Affiliations:** 1 Faculty of Medicine and Pharmacy, Medical-Pharmaceutical Research Center, “Dunarea de Jos” University of Galati, 800008 Galati, Romania; steliana.tudor@ugal.ro (S.S.T.); claudia.stefan@ugal.ro (C.S.S.); ionela.fertu@ugal.ro (I.D.F.); 2 Faculty of Medicine, Titu Maiorescu University, 031593 Bucharest, Romania; 3 Central Military Emergency University Hospital “Dr. Carol Davila”, 010825 Bucharest, Romania

**Keywords:** wearable devices, cardiovascular disease, P4 medicine, predictive medicine, preventive cardiology, personalized medicine, participatory care, digital biomarkers, artificial intelligence, remote monitoring

## Abstract

Cardiovascular diseases remain the leading cause of global mortality, yet conventional diagnostics are episodic and clinic-centered, missing the dynamic physiological events that unfold between encounters. Wearable devices offer continuous, real-world monitoring and, together with artificial intelligence, digital biomarkers, and telecardiology, increasingly support a shift toward predictive, preventive, personalized, and participatory (P4) cardiovascular care. This narrative review synthesizes the contemporary evidence base for wearable cardiovascular technology and organizes it around the four pillars of P4 medicine, with the explicit aim of distinguishing what is clinically proven from what remains aspirational. The wearable ecosystem now spans consumer smartwatches, medical-grade ECG patches, smart textiles, and emerging soft bioelectronics, generating an expanding repertoire of digital biomarkers. Evidence is strongest where validation is most mature: atrial fibrillation screening, supported by large-scale studies, and structured heart-failure telemonitoring, associated with reductions in heart-failure hospitalization of 18–32% in structured programs. For acute coronary syndrome triage, cuffless blood pressure, cardiac rehabilitation, and AI-derived prognostic markers, the supporting evidence is growing but rests largely on analytical and early clinical validation rather than on demonstrated improvements in hard cardiovascular outcomes. Across all four pillars, translation is constrained by accuracy variability across demographic subgroups, regulatory fragmentation, data privacy concerns, interoperability deficits, and inequitable access for elderly, low-income, and low- and middle-income populations. Realizing the P4 promise will require harmonized validation standards, demographic-stratified accuracy reporting, equitable access strategies, and a clinical infrastructure capable of converting continuous wearable data into actionable decisions.

## 1. Introduction

Cardiovascular diseases (CVDs) remain the leading cause of morbidity and mortality worldwide, accounting for an estimated 17.9 million deaths annually and representing approximately 32% of all global deaths [1]. Coronary artery disease, heart failure, stroke, cardiac arrhythmia, and hypertension collectively impose an enormous clinical and economic burden on healthcare systems at all income levels, with a prevalence projected to rise further, driven by aging populations, urbanization, and the global epidemic of metabolic risk factors [2]. Despite substantial advances in pharmacotherapy, interventional cardiology, and cardiac surgery, a critical diagnostic gap persists; many life-threatening cardiovascular events occur in individuals with unrecognized or inadequately monitored disease. Paroxysmal arrhythmias, silent myocardial ischemia, asymptomatic left ventricular dysfunction, and subclinical hypertension frequently evade detection within the episodic framework of conventional clinical care, with the consequences including delayed treatment initiation, preventable hospitalizations, and avoidable mortality [3,4].

Traditional cardiovascular diagnostics are inherently episodic and clinic-centered. The standard 12-lead electrocardiogram (ECG), resting echocardiography, and point-in-time laboratory biomarkers each capture a momentary physiological snapshot, inevitably missing events that occur between clinical appointments. The 24 to 48 h Holter monitor, long considered the standard of ambulatory cardiac monitoring, demonstrates diagnostic yields of only 15–39% for paroxysmal arrhythmias, owing to its limited recording window [5]. Extended event monitors and traditional ambulatory blood pressure systems impose logistical burdens that restrict their use to selected high-risk populations. The fundamental limitation is structural. A reactive care model in which patients present with symptoms and then undergo investigation is misaligned with the pathophysiology of CVD, where subclinical disease evolves silently for years before manifesting as an acute event [4,6].

The past decade has witnessed a transformative proliferation of wearable sensing technology, driven by advances in microelectronics, wireless communication, battery miniaturization, and artificial intelligence (AI). Wearable devices, defined as miniaturized electronic systems capable of continuously collecting, processing, and transmitting physiological data, have transitioned from consumer fitness gadgets to clinically relevant monitoring platforms [3,7,8]. Their clinical potential has been demonstrated at scale: the Apple Heart Study, enrolling over 419,000 participants, established the feasibility of population-level atrial fibrillation (AF) screening using a consumer smartwatch, with the Fitbit Heart Study subsequently demonstrating a positive predictive value of 98.2% for PPG-based rhythm detection when confirmed by an ECG patch [9,10]. Concurrently, deep learning algorithms applied to wearable-derived ECG and PPG signals have achieved cardiologist-level accuracy in arrhythmia classification, AF detection during sinus rhythm, and screening for asymptomatic left ventricular dysfunction [11,12,13]. Cardiovascular monitoring is migrating from the clinic to the continuum of daily life.

The convergence of wearable technology with AI-powered analytics, digital biomarkers, and telecardiology platforms supports the emergence of a new model of cardiovascular care: predictive, preventive, personalized, and participatory (P4) medicine, in which disease is anticipated before clinical manifestation, treatment is dynamically adapted to individual physiological trajectories, and the patient becomes an active co-manager of their own cardiovascular health [6,14]. The four pillars of this framework provide the organizing thread of the present review. Predictive care uses continuous data and AI to anticipate events before they occur; preventive care intervenes on risk continuously rather than episodically. Personalized care tailors monitoring and thresholds to the individual rather than to population averages; participatory care repositions the patient from a passive recipient to an active partner.

The central argument of this review is that these four pillars are not advancing in unison. Each rests on a different maturity of evidence, ranging from applications that are already clinically validated and guideline-acknowledged to others that remain supported only by analytical performance or observational association. Atrial fibrillation screening and structured heart-failure telemonitoring have reached clinical validation and, in defined scenarios, demonstrated utility. By contrast, cuffless blood pressure, AI-derived prognostic biomarkers such as vascular age indices, and digital-twin approaches remain predominantly aspirational. Rather than cataloguing devices and studies exhaustively, this review, therefore, maps the wearable cardiovascular landscape onto the four pillars of P4 medicine and, for each, distinguishes what is proven from what is promised. This framing is intended to convert a broad and heterogeneous field into a structured, critical account of where continuous cardiovascular monitoring genuinely changes care and where it does not yet do so.

To make this contribution explicit, Table 1 positions the present review against representative recent reviews on wearable cardiovascular technologies, contrasting their focus, organizing framework, and approach to evidence appraisal.

**Figure 1:**
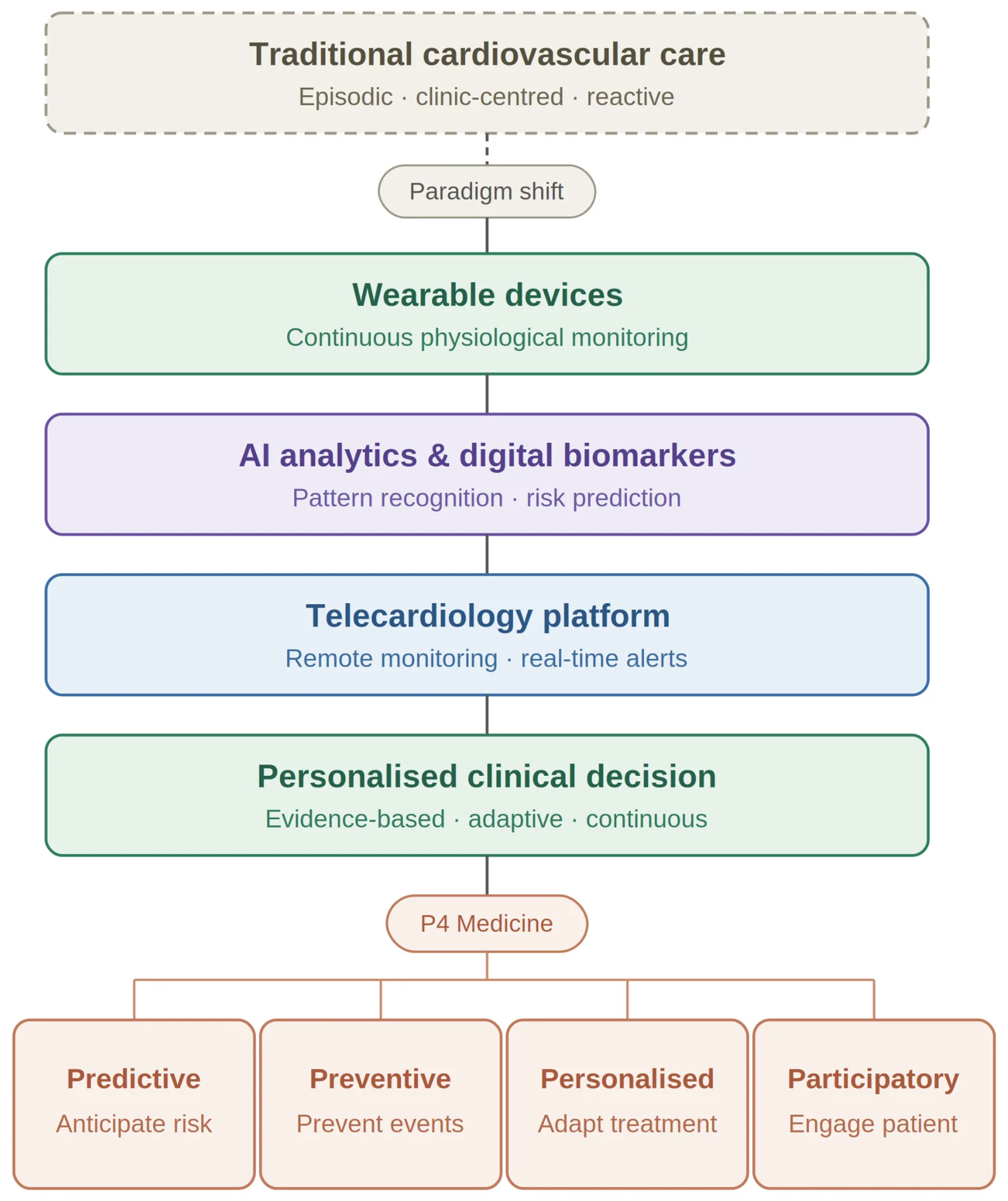
Conceptual framework: transition from traditional episodic cardiovascular care to wearable-integrated P4 medicine.

This conceptual transition from episodic cardiovascular assessment to wearable-integrated P4 medicine is summarized in Figure 1.

Wearable cardiovascular devices and their integration with artificial intelligence have been the subject of numerous recent narrative, systematic, and scoping reviews. Most either catalogue devices, signals, and reported performance or present the transition toward P4 medicine as a broadly accomplished shift [18,20,21]. The distinct contribution of the present review is that it does not treat the four pillars as equally mature. Instead of an inventory, it provides a maturity-differentiated appraisal that maps the evidence onto each pillar and, for every pillar, explicitly separates what is clinically proven from what remains aspirational. This proven-versus-aspirational framing, applied consistently across prediction, prevention, personalization, and participation, is intended to give clinicians and health-system decision-makers an actionable map of where continuous cardiovascular monitoring already changes care and where it does not yet do so.

### Literature Identification and Selection

This article is a narrative review, prepared following established guidance on narrative review methodology and reporting [22,23,24]. The literature discussed was identified through searches of PubMed, Scopus, Web of Science, and ScienceDirect, complemented by hand searching of reference lists and key society documents, for work published predominantly between 2015 and 2026. Search terms combined the concepts of wearable or consumer monitoring (for example “wearable,” “smartwatch,” “ECG patch,” “photoplethysmography”), cardiovascular conditions (“atrial fibrillation,” “heart failure,” “acute coronary syndrome,” “hypertension,” “arrhythmia”), and enabling technologies (“artificial intelligence,” “deep learning,” “digital biomarker,” “telecardiology,” “P4 medicine”). A representative search string, adapted to the specific syntax of each database, combined these three concept blocks with Boolean operators, for example (“wearable” OR “smartwatch” OR “ECG patch” OR “photoplethysmography” OR “wearable sensor”) AND (“cardiovascular” OR “atrial fibrillation” OR “heart failure” OR “acute coronary syndrome” OR “hypertension” OR “arrhythmia”) AND (“artificial intelligence” OR “deep learning” OR “machine learning” OR “digital biomarker” OR “telecardiology” OR “P4 medicine”). Searches were restricted to English-language, peer-reviewed publications, and the records were screened by title and abstract for relevance to one or more of the four pillars. Conference abstracts, preprints, and other non-peer-reviewed sources were generally excluded. Studies were selected purposively for their relevance to the four pillars of P4 cardiovascular care and their illustrative or evidentiary value, with priority given to landmark and original studies, validation studies, regulatory and professional-society consensus documents, and recent syntheses, rather than through a predefined systematic protocol. The aim is interpretive synthesis and critical appraisal rather than exhaustive or quantitative coverage. The final corpus comprises the 89 sources cited throughout; no formal record count, risk-of-bias assessment, or quantitative pooling was performed, consistent with the narrative design.

## 2. Wearable Devices and Digital Biomarkers

The predictive, preventive, personalized, and participatory model rests on a foundation of continuous sensing. Before examining what wearable data can contribute to each pillar, this section characterizes the devices that generate that data, the physiological parameters they capture, and the digital biomarkers derived from them, together with the validation status that determines how far each can currently be trusted.

### 2.1. Operational Definitions

Throughout this review, several terms recur with specific technical meanings. Table 2 provides operational definitions to support consistent interpretation, distinguishing in particular the three tiers of digital biomarkers and the successive stages of validation that recur in later sections.

### 2.2. Classification of Cardiovascular Wearable Devices

The classification adopted here is organized along two explicit axes rather than by form factor alone: regulatory and validation status (consumer or wellness versus medical grade) and signal-acquisition modality and form factor. This two-axis basis is used consistently throughout this section, and representative devices are listed in Table S1 (Supplementary Materials). Implantable loop recorders (ILRs), although the highest-fidelity continuous-monitoring tier, are implantable rather than body-worn and are, therefore, not wearable devices in the strict sense; they are retained here only as a reference comparator and are analyzed separately, never pooled with wrist-worn or patch devices.

The consumer versus medical-grade distinction is clinically decisive because it tracks the regulatory and validation gap: consumer devices are generally cleared under wellness exemptions without clinical data, whereas medical-grade devices undergo formal validation [3,25]. The two axes, form factor and regulatory tier, together determine both the signals a device captures and how far its outputs can be trusted clinically, which is why the same measurement (for example, single-lead ECG) carries different evidential weight depending on the device class [3,25,26].

**Figure 2:**
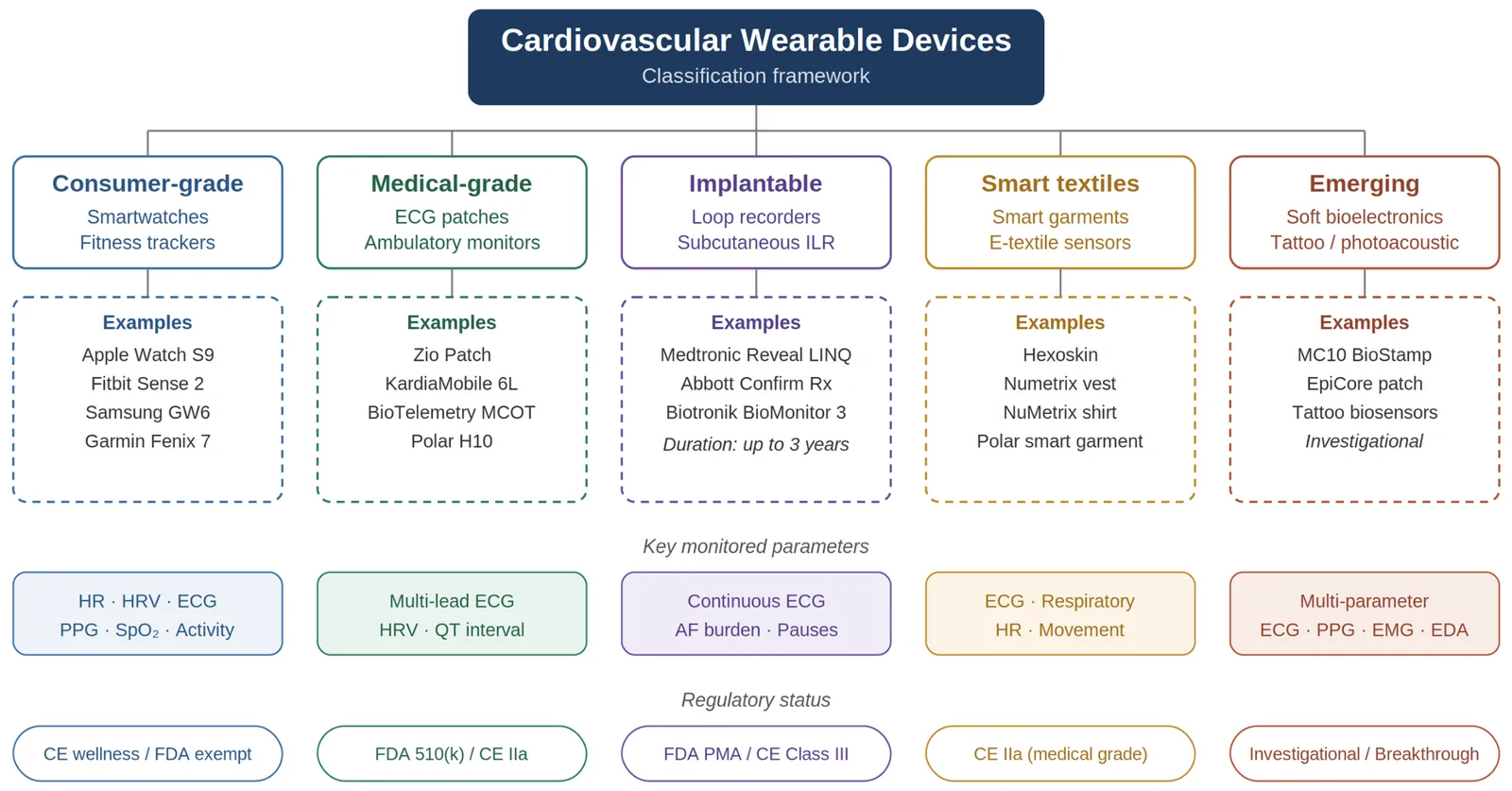
Classification of cardiovascular wearable devices by category, monitored parameters, and regulatory status.

The device classification is illustrated in Figure 2 and detailed in Table S1.

#### 2.2.1. Consumer-Grade Wearables: Smartwatches and Fitness Trackers

Consumer-grade wearables (smartwatches and fitness trackers) are the most widely deployed, offering PPG heart rate, single-lead ECG, SpO2, and activity tracking at scale, but most operate under wellness exemptions with an accuracy that degrades during motion and arrhythmia [3,27]. Their reach makes them the principal vehicle for population screening and the participatory pillar, despite limited individual-level diagnostic validation [9,10].

#### 2.2.2. Medical-Grade ECG Patches and Ambulatory Monitors

Medical-grade ambulatory ECG devices (adhesive patches and mobile cardiac telemetry) undergo formal regulatory clearance and approach Holter-equivalent arrhythmia detection over multi-day recording, making them the reference for wearable rhythm diagnosis [26,28]. They bridge consumer convenience and clinical rigor but require a prescription and are less suited to continuous long-term population use [26].

#### 2.2.3. Implantable Loop Recorders (Reference Comparator)

Implantable loop recorders represent the highest-fidelity long-term rhythm monitoring, detecting AF at several times the rate of intermittent monitoring over up to three years, and serve here as the reference comparator against which non-invasive wearables are judged [29]. Being implantable, they are not wearables in the strict sense but define the upper bound of ambulatory arrhythmia detection [7,26].

#### 2.2.4. Smart Textiles, Flexible Bioelectronics and Emerging Platforms

Smart textiles and soft bioelectronics integrate conductive fibers or printed electrodes into garments and skin patches, enabling comfortable multi-lead ECG, respiration, and activity capture, and represent the emerging frontier of unobtrusive continuous monitoring [30]. Comparative clinical validation remains limited, so they are presently research-grade or early-commercial, rather than established clinical tools [30].

### 2.3. Monitored Physiological Parameters

The physiological information accessible through cardiovascular wearables now spans electrical, optical, mechanical, and chemical signals well beyond the heart rate of first-generation trackers [27,31]. Heart rate, derivable from PPG or ECG, shows wrist-PPG mean absolute errors of 2 to 5 bpm at rest, degrading during exercise, in AF, and in darker skin tones [27]. Heart rate variability (RMSSD, SDNN, LF/HF), a non-invasive autonomic index whose reduction predicts mortality and adverse post-infarction prognosis, is now available on consumer smartwatches, though standardization across devices remains inconsistent [14,27,32].

Wearable ECG depends on electrode type and count, which determine whether a device supports single-lead rhythm screening or multi-lead morphology. Gel electrodes give the cleanest signal but irritate skin. Dry electrodes enable brief gel-free recordings. Textile electrodes allow multi-day wear at higher impedance, and capacitive electrodes sense through fabric but are motion-sensitive. Single-lead ECG suffices for rhythm classification, especially with AI augmentation [26,28,33], while medical-grade patches offer two- to six-lead configurations for QT, ST-segment, and P-wave analysis [26]. Choi et al. showed nine-lead reconstruction from smartwatch electrodes matching 12-lead diagnostic performance (AUC 0.987 vs. 0.991, *p* = 0.745) [34], and AI-enhanced protocols in young athletes achieve reliable automated risk stratification [35].

Photoplethysmography exploits differential optical absorption of oxygenated and deoxygenated hemoglobin, with its accuracy varying with optical geometry, wavelength, and physiology (melanin, perfusion, temperature, motion). Beyond heart rate, PPG enables pulse wave velocity, respiratory rate, and cuffless blood pressure via pulse transit time [31,36]; the AI-derived PPG-age metric independently associates with cardiovascular mortality [36]. SpO2 from dual-wavelength oximetry aids nocturnal-desaturation screening but is unreliable below 90%, and cuffless blood pressure remains adjunctive, with mean systolic differences of 2 to 8 mmHg, falling short of AAMI and British Hypertension Society thresholds [3,31].

Triaxial accelerometers quantify activity, step count, and sedentary time, parameters with established predictive value. Each additional 1000 daily steps associates with a 6 to 10% lower cardiovascular mortality risk, and sedentary time above 10 h daily predicts incident heart failure [14]. In children and adolescents, sedentary behavior additionally contributes to musculoskeletal and postural disorders amenable to corrective intervention, underscoring the value of early activity monitoring [37]. Actigraphy–PPG fusion identifies obstructive sleep apnea risk and nocturnal arrhythmias, and the cardiovascular exposome framework positions these longitudinal streams as an integrated tool for cumulative risk [4,14]. Because single-modality monitoring has inherent failure modes, multimodal sensor fusion (at signal, feature, or decision level) improves robustness, so that PPG screening confirmed by on-demand single-lead ECG and multiparameter heart-failure indices outperform any single parameter.

### 2.4. Digital Biomarkers: Taxonomy, Clinical Relevance and Validation

Digital biomarkers are objective, quantifiable physiological and behavioral measures collected through digital devices as indicators of biological, pathological, or pharmacological processes [32], distinguished from traditional biomarkers by continuous capture, ecological validity, and temporal granularity [32]. Three tiers are recognized: primary (directly measured: HR, HRV, PPG, ECG), secondary (computationally derived: RMSSD, pulse wave velocity, step count, SpO2 nadir), and tertiary (AI-inferred composite indices: PPG age, ECG age, AF burden, decompensation risk) [32,36]. These tertiary biomarkers are central to the predictive and personalized pillars, translating raw signals into individualized, forward-looking risk estimates.

Classical cardiovascular biomarkers (cardiac troponins I and T, NT-proBNP, BNP, hsCRP, LDL-cholesterol, HbA1c, and eGFR) provide validated guideline-endorsed anchors for diagnosis, risk stratification, and therapeutic monitoring [4]. Their principal limitation is episodic availability. A single phlebotomy snapshot cannot capture dynamic cardiovascular fluctuations between clinical encounters. Digital biomarkers complement this framework by providing the temporal dimension that classical biomarkers lack. Table 3 presents an extended comparison across twelve clinically relevant dimensions.

The integration of both biomarker classes enables the construction of a dynamic, individualized cardiovascular risk profile that neither modality could achieve in isolation. A patient with mildly elevated NT-proBNP combined with wearable-detected 20% reduction in daily step count, progressive nocturnal tachycardia, and increasing AF burden over seven days constitutes a composite decompensation signal that classic biomarkers alone would not capture [32,38].

The personalized-medicine utility of digital biomarkers derives from individual baseline characterization, detecting personally significant deviations rather than population-average threshold crossings. Wearable-derived functional trajectories individualized to each patient’s baseline outperform fixed-threshold alerts in heart failure [38,39], continuous AF-burden quantification refines anticoagulation beyond binary classification [9,10], and AI-derived ECG and PPG age quantify biological ageing with prognostic value beyond established scores [36].

Validation of a digital biomarker is best understood as a three-stage progression: analytical validation (the device measures the signal accurately and reproducibly against a reference standard), clinical validation (the measure is associated with a meaningful health state or outcome) and clinical-utility validation (acting on the measure improves outcomes beyond standard care). These stages are distinct, and a biomarker may be analytically robust yet clinically unproven.

Only a minority of wearable digital biomarkers have progressed beyond analytical validation. Those with clinical validation and at least partial clinical utility are PPG- or single-lead-ECG-based AF detection (the most mature, with population-scale clinical validation and guideline acknowledgement of AF burden), accelerometry-derived step count and physical activity (robustly associated with cardiovascular mortality and event risk), and resting heart rate. Biomarkers with clinical validation but limited utility evidence include multiparameter heart-failure decompensation indices (validated in programs, with an effect that is partly organizational) and HRV (outcome-associated but with measurement-standardization gaps). Biomarkers still predominantly at the analytical stage, and therefore not yet clinically validated for diagnosis, include cuffless blood pressure, wearable SpO2 below 90%, AI-derived PPG age and ECG age (prognostic associations only, observational), AI-ECG left-ventricular-dysfunction screening (promising but not outcome validated in wearable form), and thoracic bioimpedance. 

### 2.5. Technical Performance, Regulatory Landscape and Validation Maturity

Device accuracy varies substantially across categories, parameters, and populations. Consumer smartwatches show acceptable resting HR accuracy (MAE 2 to 5 bpm) but overestimate HR by 10 to 15 bpm during AF and underperform during exercise [27]. PPG-based AF detection achieves pooled sensitivities of 93 to 98% and specificities of 88 to 98%, though 3 to 6% false-positive rates generate substantial unnecessary downstream investigation at scale [9,10,40]. Medical-grade patches approach Holter-equivalent accuracy, while Sîngeap et al. reported an inter-study AF-detection sensitivity of 71 to 99% and a specificity of 73 to 99%, underscoring the need for standardized head-to-head validation [19].

The regulatory landscape compounds this heterogeneity. The FDA classifies wearable health technology under the Software as a Medical Device framework (Class I wellness exempt; Class II 510(k)/De Novo; Class III PMA), while EU MDR 2017/745 applies a parallel risk-based classification in which health software is generally Class IIa or IIb and ILRs are Class III [3,7,25]. A direct US-EU comparison (Table 4) exposes actionable gaps. The FDA’s wellness exemption is broader than the EU’s intended-medical-purpose test, so the same device may reach the US market without clinical data yet be regulated in the EU; the MDR notified-body bottleneck delays access; adaptive AI remains immaturely handled in both; and neither framework mandates demographically stratified validation, perpetuating the equity gap. Standardization initiatives (IEEE P11073, AAMI and British Hypertension Society protocols, EHRA and Heart Rhythm Society consensus, CDISC) are converging but incomplete, a prerequisite for moving wearables from adjunctive tools to guideline-endorsed instruments [3,25,41].

The strong accuracy figures reported throughout this review (AUC 0.87 to 0.97; AF PPV up to 98.2%) require careful interpretation because most derive from analytical validation under favorable conditions rather than prospective real-world deployment. Three caveats apply. First is dataset selection: AI-ECG models are typically trained and tested within one health system, so reported performance reflects internal rather than external validity. Second is prevalence sensitivity: positive predictive value is prevalence-dependent, so a device with 98% sensitivity and 94% specificity yields many false positives in low-prevalence screening, as in the 66% false-positive rate of the Apple Heart Study. Third is operating conditions: accuracy measured at rest degrades with motion, arrhythmia, skin tone, and body habitus. External prospective validation is, therefore, the decisive tier (Table 5). Generalizability is further limited because pivotal datasets skew younger, lighter-skinned, male, and higher-income, precisely the opposite of the highest-burden populations. The reported metrics should be read as upper bounds until externally and demographically validated.

## 3. Clinical Applications Across the Cardiovascular Spectrum

This section examines the principal clinical applications of wearable cardiovascular technology, from atrial fibrillation screening to preventive cardiology. For each, the relevant pillar of the P4 framework is identified and the maturity of the supporting evidence is assessed because it is across these applications that the four pillars are realized unevenly.

### 3.1. Atrial Fibrillation

Atrial fibrillation screening is the most mature application and the clearest example of the predictive and preventive pillars in action.

Atrial fibrillation affects an estimated 37 to 44 million people worldwide and is a leading cause of stroke, yet it is frequently paroxysmal and asymptomatic. So, conventional intermittent monitoring misses a substantial fraction of cases [8]. Wearable PPG and single-lead ECG enable continuous or on-demand screening that captures episodes outside clinical settings, and consumer devices have received regulatory clearance for irregular-rhythm notification [3,9,10]. This makes AF the flagship preventive application, where large-scale evidence for wearable screening is strongest [9,10,40,44].

A paradigm-shifting application involves AI detection of the ECG substrate of AF during periods of sinus rhythm. Attia et al. trained a convolutional neural network on 649,931 ECGs from 180,922 patients, identifying AF-associated features during sinus rhythm with AUC 0.87 to 0.90, sensitivity 79 to 82%, and specificity 79 to 83% [12]. The clinical implication is transformative. A routine 30 s sinus-rhythm smartwatch ECG could identify individuals harboring paroxysmal AF propensity before any detected episode, enabling pre-emptive anticoagulation consideration and monitoring intensification. This is the predictive pillar in its most literal form, anticipating a condition before it manifests. Systematic reviews of AI-based atrial fibrillation detection report consistently high analytical accuracy for deep-learning models applied to single-lead and wearable recordings, while noting that validation remains predominantly retrospective [45,46,47].

Three landmark studies anchor consumer wearable AF screening. The Apple Heart Study (419,297 participants) showed that irregular-pulse notifications had a positive predictive value of 0.84 against ECG-patch confirmation, though only 0.5% received notifications, and false positives were common in low-prevalence groups [9]. The Fitbit Heart Study (455,699 participants) reported a PPV of 98.2% for software-confirmed episodes [10]. The Huawei study and the mAFA-II randomized trial extended these findings to integrated screen-and-manage pathways with improved outcomes [40]. Together, they establish population-scale feasibility while exposing the false-positive burden of unselected screening.

Taken together, the AF evidence supports validated use of consumer PPG and single-lead ECG for opportunistic screening and AF-burden estimation, which already informs anticoagulation decisions in defined scenarios; what remains unproven is reliable diagnosis in unselected low-prevalence populations, performance in patients with a high ectopy burden, and the prediction of AF during sinus rhythm outside single-institution datasets.

Beyond AF, wearables detect premature atrial and ventricular complexes, supraventricular tachycardia, and pauses, though evidence here is less mature and largely observational [19,26]. Signal quality, motion artifact, and the absence of a full 12-lead morphology limit definitive characterization, so wearable findings typically trigger confirmatory ambulatory monitoring rather than standalone diagnosis [26]. The clinical value lies in extending the observation window and capturing infrequent episodes that short in-clinic recordings miss.

### 3.2. Heart Failure

Heart-failure monitoring is the second most mature application and the clearest expression of the personalized pillar, since it depends on tracking deviations from an individual patient’s own baseline.

Heart failure affects over 64 million individuals globally, with recurrent acute decompensation accounting for most of the HF-related healthcare expenditure [1,48]. Wearable devices enable hemodynamic surveillance between clinical encounters, detecting fluid accumulation, functional decline, and autonomic deterioration that precedes symptomatic decompensation by days to weeks [38,39]. Key monitored parameters include resting HR and HR trends, daily step count, nocturnal HR, SpO2, sleep quality, and body weight via connected smart scales [38,48]. The EHRA 2022 Cardiovascular Round Table endorsed wearable-based remote monitoring as a validated component of HF disease management programs, citing evidence of 15 to 30% reductions in 30-day readmission rates in structured telemonitoring trials [41,49].

Multiparameter AI models substantially improve decompensation prediction beyond single-parameter approaches [50,51]. Devin et al. documented AI models integrating HR, activity, sleep, and weight data achieving AUC 0.78 to 0.92 for hospitalization prediction within 7 to 14 days, compared with 0.63 to 0.71 for single-parameter approaches [38]. The implementation framework recommended by Pizarro et al. employs a tiered alert system, with green (stable), yellow (early warning, nurse contact), and red (urgent clinical review), driven by objective wearable data rather than patient-reported symptoms [39].

Thoracic bioimpedance, which tracks fluid accumulation preceding overt decompensation, and multiparameter wearable indices have shown lead times of 7 to 14 days before heart-failure hospitalization [38]. Implantable pulmonary-artery pressure monitoring (CardioMEMS) remains the most outcome-validated remote HF technology, but wearable, non-invasive alternatives are advancing [52]. The demonstrated benefit is partly organizational, since alerts require a structured response team to translate into avoided admissions, which is why effect sizes vary with the program design [38,39].

### 3.3. Acute Coronary Syndromes

Wearable ECG in acute coronary syndromes serves the predictive pillar at the point of acute presentation, offering earlier rhythm capture outside the hospital.

Prompt ECG acquisition is critical in acute coronary syndrome, where the time to diagnosis drives outcome. Smartwatch and patch ECG can shorten the interval to first medical contact by capturing ST-segment changes at symptom onset [34]. Choi et al. demonstrated nine-lead reconstruction from a smartwatch approaching 12-lead diagnostic performance in a small cohort [34], but sensitivity for posterior infarction and reliability outside controlled settings remain unproven, so wearable ECG is currently a triage adjunct rather than an approved ACS diagnostic tool.

Telemetry-enabled smartwatch ECG transmission to receiving cardiologists has been piloted in several European health systems as a supplement to ambulance-based 12-lead ECG, with preliminary feasibility data for STEMI identification in most transmissions [28,34]. In low- and middle-income countries, where standard 12-lead ECG availability is limited, smartwatch ECG combined with telemedicine platforms offers a scalable diagnostic accessibility solution with significant public health potential [53]. Important limitations constrain the clinical role. Single-lead ECG cannot localize infarct territory or assess the reciprocal changes that are essential for equivocal STEMI adjudication. AI-ACS algorithms exhibit lower sensitivity for posterior MI, LBBB-masked STEMI, and Wellens’ syndrome patterns [19,28], and current regulatory approvals extend only to rhythm classification, not ACS diagnosis. Smartwatch ECG should, therefore, be positioned as an adjunctive accessibility tool pending further prospective validation.

### 3.4. Hypertension

Cuffless blood pressure monitoring targets the preventive and personalized pillars, though it remains the least validated of the major applications.

Hypertension affects approximately 1.28 billion adults globally and is the leading modifiable cardiovascular risk factor, yet clinic measurement misses masked and nocturnal hypertension [3]. Cuffless wearable blood pressure, via pulse transit time or pulse-wave analysis, promises continuous ambulatory profiling that could personalize therapy [31]. This targets the preventive and personalized pillars, capturing patterns invisible to episodic cuff measurement.

The validation landscape remains heterogeneous and incompletely resolved. No cuffless consumer wearable BP device had, as of 2026, completed full validation against all applicable clinical protocols across diverse populations, including pregnant women, elderly individuals, and AF patients, the groups in whom accurate BP measurement is most consequential [3,31]. Iqbal et al., in a comprehensive review of 23 validation studies, documented the mean differences between cuffless devices and the reference sphygmomanometer of 0.3 to 7.8 mmHg for systolic BP and 0.2 to 8.6 mmHg for diastolic BP [31], below the AAMI SP10 and European Society of Hypertension protocol thresholds required for clinical guideline endorsement. Current devices serve a valuable adjunctive role in trend monitoring and ambulatory BP profiling while formal validation is completed.

### 3.5. Cardiac Rehabilitation and Secondary Prevention

Cardiac rehabilitation is where the participatory pillar is most evident, since wearables enable patients to conduct supervised rehabilitation in their own environment.

Cardiac rehabilitation reduces all-cause mortality and hospitalization but is chronically underused, with participation often below 30% because of travel, scheduling, and access barriers [54]. Wearable-enabled home-based rehabilitation addresses these barriers directly. Remote HR, activity, and ECG monitoring allow supervised exercise outside facilities, and randomized evidence shows home-based programs achieve peak-VO2 gains and completion rates comparable to or exceeding facility-based care [54,55]. This is the clearest expression of the participatory pillar, where the outcome is engagement and adherence rather than diagnostic accuracy [48,54]. In the related domain of sports and exercise monitoring, machine learning integration of anthropometric, physiological, and biological data has enabled multiparameter profiling of elite youth athletes, illustrating how wearable and laboratory data can be combined for individualized physiological assessment [56].

Following acute MI or PCI, serial step count tracking, HR recovery profiling, and HRV trajectory assessment provide early insight into cardiovascular recuperation and guide progressive activity recommendations [4,7]. Wearable-detected reduced HRV at 30 days post-MI predicts adverse 12-month outcomes, offering a non-invasive stratification tool for risk-adapted follow-up intensity [14]. Post-cardiac surgery patients benefit from continuous ECG patch surveillance for postoperative AF, occurring in 20 to 40% of cases, enabling prompt treatment initiation and reduction in perioperative stroke risk [26]. In primary and secondary prevention, wearable sleep monitoring identifies obstructive sleep apnea through nocturnal SpO2 desaturation patterns and fragmented sleep architecture, enabling referral for formal polysomnography and CPAP evaluation in this prevalent, underdiagnosed cardiovascular risk amplifier [4].

### 3.6. Arrhythmia Monitoring Beyond Atrial Fibrillation

Ventricular arrhythmias account for approximately 50% of cardiovascular mortality via sudden cardiac death (SCD) [7,26]. Wearable ECG devices with sufficient signal fidelity and AI morphology analysis can detect ventricular ectopy burden, non-sustained VT episodes, and QT prolongation in ambulatory settings [26,57,58]. Srivats et al. reviewed emerging wearable algorithms for SCD risk stratification in inherited arrhythmia syndromes, including Brugada syndrome, long QT syndrome, and hypertrophic cardiomyopathy, documenting early evidence for ambulatory risk profiling that could guide defibrillator implant decisions [57].

Consumer smartwatches detect paroxysmal SVT, including AVNRT, AVRT and atrial tachycardia, through HR surge detection combined with single-lead ECG morphology; the combination of abrupt-onset tachycardia, a regular narrow-complex pattern, and patient symptom logging provides clinically useful triage information for electrophysiology referral [26,28]. Pre-excitation patterns may be detectable in high-fidelity single-lead ECG patch recordings, enabling Wolff–Parkinson–White identification in asymptomatic young patients [26]. Post-ablation surveillance, requiring sustained monitoring to assess procedural success and detect early recurrence, benefits substantially from wearable ECG patches, providing 14- to 30-day continuous recording and achieving a 2- to 4-fold higher recurrence detection than the standard 24 h Holter, with the EHRA digital consensus advocating systematic post-ablation wearable monitoring at 3, 6, and 12 months as a quality benchmark [26,42]. In pediatric and adult congenital heart disease populations, ECG patches with pediatric-adapted sizing and AI algorithms trained on pediatric datasets enable non-invasive ambulatory monitoring aligned with grown-up congenital heart disease (GUCH) guideline requirements [7,26].

### 3.7. Preventive Cardiology and Risk Factor Surveillance

Preventive cardiology is the broadest expression of the preventive and participatory pillars, extending monitoring from patients to at-risk but asymptomatic individuals.

Physical inactivity is a leading modifiable cardiovascular risk factor, and accelerometry-derived activity monitoring is robustly, dose-dependently associated with reduced cardiovascular mortality, with each additional 1000 daily steps lowering risk by 6 to 10% [14]. Consumer wearables promote activity through feedback, goal-setting, and social features, and large cohorts link device-measured activity to hard outcomes [14]. This positions physical-activity monitoring as one of the most evidence-backed preventive applications, spanning the preventive and participatory pillars.

Wearable HRV monitoring provides an objective physiological correlate of chronic psychosocial stress and reduced parasympathetic tone (low RMSSD, reduced HF power), reflecting sustained sympathetic dominance associated with burnout, depression, and adverse cardiovascular outcomes [14,27]. Longitudinal HRV monitoring enables the detection of sustained autonomic dysregulation preceding clinical cardiovascular events and provides an objective outcome metric for stress reduction interventions [27]. Combining wearable-generated objective physiological data with validated patient-reported outcome instruments, such as the SF-36 for quality of life and the PHQ-9 for depressive symptom burden [59], offers a route to a more comprehensive cardiovascular and psychological health profile than either data source alone; wearable behavioral digital biomarkers have been shown to track patient-reported physical limitation, symptom burden, and quality of life in cardiovascular patients [60]. The synthesis of wearable-derived activity, sleep, HRV, HR, rhythm, and body composition data into integrated lifestyle cardiovascular risk profiles constitutes the preventive cardiology application with potentially the broadest population health impact. Pedroso and Khera articulated an AI-enhanced consumer device-based cardiovascular screening framework in which composite wearable-derived risk indices identify asymptomatic high-risk individuals for targeted preventive intervention, extending preventive cardiology reach from clinic to community [61]. Reduced HRV and elevated arrhythmic burden independently associate with depression and quality-of-life impairment, while chronic stress amplifies the arrhythmogenic substrate, a bidirectional cycle that wearable HRV and rhythm monitoring is positioned to detect and interrupt [62].

The principal clinical studies supporting these applications are summarized in the Supplementary Materials (Table S2, study design and characteristics; Table S3, primary outcomes, diagnostic accuracy, and level of evidence).

## 4. Artificial Intelligence and Telecardiology as Enablers

If wearable devices provide the sensing foundation of P4 cardiovascular care, artificial intelligence and telecardiology are the layers that make the four pillars operational. AI converts raw signals into predictive and personalized indices, while telecardiology provides the organizational infrastructure through which those indices reach clinical decisions and engage the patient. This section examines both, together with their limitations.

### 4.1. Artificial Intelligence in Wearable Cardiology

#### 4.1.1. Machine Learning, Deep Learning and ECG-Based Applications

AI applied to wearable cardiovascular data spans several model families: convolutional neural networks for ECG and PPG morphology, recurrent and LSTM networks for temporal trends, and ensemble methods for multimodal risk [11,12,13,63,64]. The landmark demonstration is AI detection of AF from sinus-rhythm ECG (AUC 0.87 to 0.90), which infers latent atrial substrate invisible to human readers [12], alongside AI-ECG screening for left-ventricular dysfunction (AUC 0.93) [13] and 12-class arrhythmia classification matching cardiologists (AUC 0.97) [11]. Beyond rhythm classification, AI-ECG has been extended to the detection of electrolyte disturbance, aortic stenosis, and myocardial infarction from reduced-lead recordings [65,66,67], and systematic reviews of AI-assisted electrocardiography confirm reproducible diagnostic gains in outpatient and perioperative settings [68,69,70]. These wearable applications sit within a broader cardiovascular AI landscape in which deep learning already achieves expert-level performance in echocardiographic interpretation, coronary CT analysis, and calcium scoring, and in which machine learning improves prognostic stratification in suspected coronary artery disease [71,72,73,74,75]. The wearable domain inherits both the methods and the external-validation challenges of that literature. These establish that AI extracts clinically meaningful information beyond conventional interpretation, but most are retrospective, single-institution, and not yet outcome-validated in wearable form, a recurring pattern across the predictive pillar [11,12,13].

#### 4.1.2. PPG-Based AI, Multimodal Fusion, Explainability and Federated Learning

PPG-based AI diagnostics extend beyond AF to blood-pressure estimation, vascular ageing, and decompensation prediction, with the AI-derived PPG-age metric independently predicting cardiovascular mortality [36]. Multiparameter models fusing HR, activity, SpO2, and weight forecast heart-failure decompensation with 7- to 14-day lead times [38]. Across these applications the pattern is consistent: strong analytical performance on curated data, but sparse external validation and demographic representativeness. So, real-world accuracy is likely lower than headline figures suggest [19,36,38].

#### 4.1.3. Critical Appraisal and Limitations of AI in Wearable Cardiology

Deep learning carries specific limitations beyond its accuracy headlines (Tables S4 and S5). For data dependence and overfitting, models fit dataset-specific artifacts and spurious correlates, so internal accuracy overstates external performance and AUCs without external validation are upper bounds. For distribution shift, performance degrades when the deployment population, device generation or signal quality differ from training, a recurrent wearable problem. Opacity impedes error analysis, regulatory scrutiny and trust. For brittleness, small perturbations or motion artifact change outputs disproportionately. For label dependence, models inherit their training labels’ biases. Calibration, essential for threshold-based alerts, is rarely reported. Wearable AI studies should, therefore, report external validation, calibration, subgroup performance and signal-quality coverage as standard.

Ethical and governance issues are intrinsic to clinical AI and not resolved by accuracy alone [76]. For algorithmic fairness, models trained on non-representative data underperform in under-represented groups, risking mis-triage of the highest-burden populations, so fairness-constrained training and stratified reporting are required. For transparency and accountability, opaque outputs complicate consent and responsibility when an alert is wrong, making explainability and human-in-the-loop accountability prerequisites. For data autonomy, continuous data enable inference of sensitive states (stress, sleep, location) that consent frameworks rarely cover. For automation bias and access, over-reliance can erode clinical judgement while benefits accrue to digitally connected populations. AI in wearable cardiology is, therefore, clinically promising but ethically and methodologically immature, requiring governance commensurate with its reach.

### 4.2. Telecardiology and Clinical Integration

#### 4.2.1. Telecardiology Architecture and Integration Models

Telecardiology provides the organizational framework that turns wearable data into care through remote monitoring, virtual visits, and clinician dashboards that integrate device streams into a workflow [5,25,77]. Its value depends less on the sensor than on the response system; structured teams, defined alert thresholds, and clear escalation pathways determine whether data change outcomes [25,41]. Without this infrastructure, wearable data generate alert fatigue rather than benefit, which is why the participatory and personalized pillars are as much organizational as technological achievements [5,25].

#### 4.2.2. Clinical Outcomes and EHR Interoperability

Evidence from randomized trials and large cohorts shows that structured remote-monitoring programs reduce heart-failure hospitalization by 18 to 32% and improve AF management when embedded in coordinated pathways [38,39,40]. The benefit is consistently tied to the care model rather than the device alone, and the effect sizes vary with the program design, staffing, and adherence [39,54]. This positions telecardiology as the enabling layer for P4 translation, and its uneven implementation is a principal reason the promise outpaces the proof [25,78].

**Figure 3:**
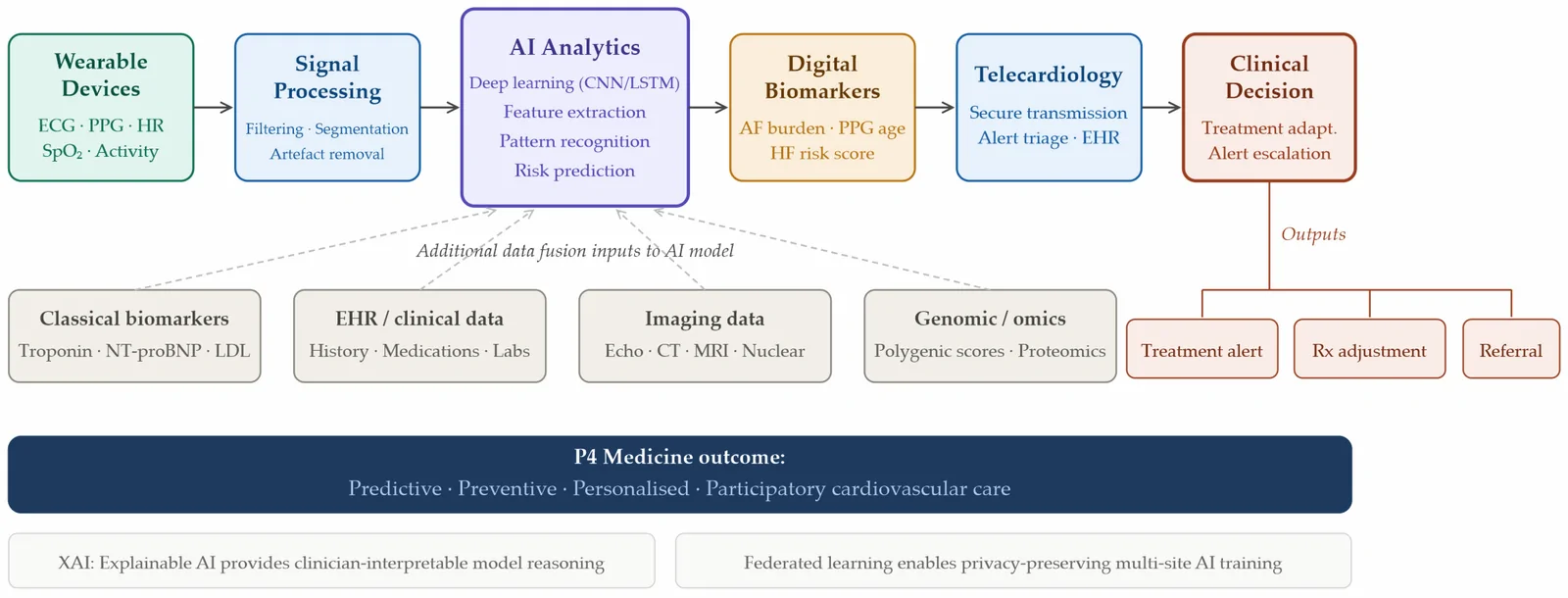
Artificial intelligence and telecardiology integration pipeline: from wearable data acquisition to personalized clinical decision.

The proposed integration pathway linking wearable data acquisition, AI analysis, telecardiology infrastructure, and clinical decision-making is illustrated in Figure 3.

## 5. Mapping the Evidence onto the Four Pillars

This section is the analytic core of the review. Rather than restating the applications, it maps the accumulated evidence onto the four pillars of P4 medicine and, for each, states plainly what is proven and what remains aspirational. The purpose is to replace an undifferentiated impression that wearables deliver predictive, preventive, personalized, and participatory cardiovascular care with a graded account of where each pillar actually stands.

### 5.1. Cross-Cutting Tensions in the Evidence

To convert the preceding application sections from description into structured comparison, Tables S6 and S7 (Supplementary Materials) summarize, for each major application, the device or modality, monitored signals, AI algorithm class and features, representative performance, validation tier and level of evidence, and the key finding and limitation. Each metric is reported alongside its reference standard and population so that heterogeneity is visible rather than hidden; the figures are drawn entirely from studies discussed in this review and are not pooled estimates.

Comparison across studies reveals several tensions that a narrative reading can obscure. First, headline AF-detection metrics diverge widely (sensitivity 71 to 99%, specificity 73 to 99%), largely because reference standards and populations differ (single-lead patch versus 12-lead ECG, screening versus enriched cohorts), rather than because devices truly differ by that margin. The Apple Heart Study PPV of 0.84 and the Fitbit PPV of 0.98 are not contradictory but reflect different confirmation pathways. Second, AI-ECG results are almost uniformly retrospective and single-institution, so the strong AUCs (0.87 to 0.97) co-exist with largely untested external validity. Third, cuffless blood pressure shows a consistent contradiction between favorable manufacturer-reported agreement and independent validation that fails AAMI and ESH thresholds. Fourth, heart-failure telemonitoring trials report hospitalization reductions across a broad range (11 to 32%) that correlates less with the device than with the surrounding clinical-service model, a key gap, since the intervention effect is organizational as much as technological. These tensions are the reason this review assesses each pillar by its maturity of evidence rather than by headline performance.

Across the AF-screening studies discussed (five studies; over 1.3 million participants), PPG-based detection clustered at a sensitivity of 93 to 98% and a specificity of 88 to 98%, with a positive predictive value ranging from 0.84 to 0.98 depending on confirmation pathway and population prevalence. AI-ECG detection of AF during sinus rhythm clustered at an AUC of 0.87 to 0.97. These are descriptive ranges, not weighted pooled estimates, and the spread is driven principally by differences in reference standard and population enrichment rather than by device capability.

### 5.2. The Predictive Pillar

Predictive care is the pillar with the widest gap between promise and proof. Its clearest realized instance is AI detection of AF during sinus rhythm, which anticipates a latent condition before any episode [12]. Multiparameter heart-failure decompensation models that forecast hospitalization 7 to 14 days ahead are a second [38]. Both are genuinely predictive, but both rest largely on retrospective, single-institution data and lack prospective external validation. AI-derived prognostic biomarkers such as PPG age and ECG age predict cardiovascular mortality in large cohorts [36], yet these are observational associations rather than actionable, outcome-validated tools. Realizing the predictive promise at scale will also depend on a large-scale data analytics infrastructure capable of turning population-level signals into individual risk estimates [79]. The predictive pillar is, therefore, analytically strong and clinically aspirational. The algorithms exist and perform well internally, but evidence that acting on their predictions changes outcomes is still largely absent. Recent 2025 work reinforces this. AI-derived photoplethysmographic vascular-age indices [36] and consumer-device screening frameworks [61] remain prognostic rather than outcome-validated.

### 5.3. The Preventive Pillar

Prevention is the pillar with the most solid population-level evidence, concentrated in two areas. AF screening at scale, validated in trials enrolling nearly one million participants, enables earlier detection and, in defined scenarios, earlier anticoagulation [9,10,40]. Accelerometry-derived physical-activity monitoring is robustly and dose-dependently associated with cardiovascular risk reduction [14]. Beyond these, preventive applications such as sleep-disordered-breathing triage, psychosocial-stress detection, and integrated lifestyle risk profiling are conceptually compelling and supported by association studies but not yet by preventive-outcome trials. Prevention is thus the pillar where wearables come closest to a demonstrated population benefit, provided the screening is embedded in a confirmation and referral pathway rather than delivered as an isolated notification Recent syntheses position consumer-device data within scalable population screening and prevention pathways [20,61].

### 5.4. The Personalized Pillar

Personalization is the pillar most native to wearable data because continuous monitoring characterizes an individual baseline that episodic testing cannot. Its strongest realized instance is heart-failure management, where individualized functional-capacity trajectories outperform fixed-threshold alerts [38,39], and AF-burden quantification refines anticoagulation beyond binary classification [29,42]. The tertiary, AI-inferred digital biomarkers described earlier are the technical substrate of this pillar [80,81]. The limitation is that personalization is validated mainly within recent structured heart-failure remote-monitoring studies [38,39], exemplifying individualized, baseline-referenced management. For programs and specific conditions, a general capacity to tailor cardiovascular care to each patient’s wearable-defined baseline, across conditions, remains partial. Personalization is, therefore, clinically real in heart failure and atrial fibrillation and promising but unproven elsewhere.

### 5.5. The Participatory Pillar

Participation is the pillar least amenable to conventional diagnostic-accuracy evidence because its outcomes are engagement, adherence, and self-management rather than sensitivity and specificity. Wearables demonstrably increase patient engagement. Remote cardiac rehabilitation achieves higher completion than facility-based programs [54], and patients who observe their own physiological responses show improved adherence and self-efficacy [48,54]. Hybrid patient-initiated monitoring models formalize this active role [39,41]. The unresolved tension is equity. Participation benefits the digitally connected and can widen disparities for the elderly, low-income, and low- and middle-income populations who carry the greatest cardiovascular burden. The participatory pillar is thus behaviorally validated but structurally constrained, and its promise depends on deliberate equity design rather than on technology alone.

### 5.6. A Consolidated Picture

Taken together, the four pillars occupy different points on a single maturity gradient. Prevention (AF screening, physical activity) and personalization (heart failure, AF burden) rest on the firmest clinical and, in places, utility evidence. Prediction is analytically advanced but clinically aspirational; participation is behaviorally supported but limited by equity. This graded reading, rather than a uniform claim that wearables already deliver P4 medicine, is the central contribution of this review. The broader corpus of AI-ECG and digital-health studies that underpins these conclusions is summarized in Table S8 (Supplementary Materials), which documents, by application domain, the additional studies contributing to the evidence base and corroborates the consistent pattern of analytical strength, validation heterogeneity, and demographic underrepresentation observed throughout.

This maturity gradient can be framed through the lens of integrated health technologies, in which the four pillars are advanced not by isolated devices but by technologies that combine validated sensing, analytics, and clinical workflow integration. The wearable landscape can be pictured as a pyramid, or an inverted funnel: many devices and digital biomarkers enter at the base with analytical validation alone, progressively fewer accumulate clinical and then clinical-utility validation, and only those reaching the top, currently a small set exemplified by atrial-fibrillation screening and structured heart-failure telemonitoring, are mature enough to be integrated across all four pillars of P4 medicine simultaneously. Most technologies presently occupy the lower and middle tiers, contributing to one or two pillars rather than the full model. This framing reinforces the central message of the review: full P4 integration is currently the exception rather than the rule, and moving a technology upward through the pyramid, from analytical promise to demonstrated outcome benefit, is precisely the translational work that remains.

## 6. Barriers and Future Prospects

The graded reading of the four pillars set out above raises an obvious question: what prevents the less mature pillars from advancing, and what would move them forward? This section addresses both. It first synthesizes the barriers that constrain the transition to P4 cardiovascular care, then outlines the technological and research directions most likely to close the gap between analytical promise and clinical proof.

### 6.1. Barriers to the P4 Transition

#### 6.1.1. Accuracy, Signal Quality and Population Variability

The fundamental technical limitation of wrist-worn PPG is that signal quality degrades with motion, poor peripheral perfusion, darker skin tones, and arrhythmia, precisely the conditions and populations where monitoring matters most [27,31]. Green-wavelength PPG is robust for resting heart rate but weaker for SpO2 and cuffless blood pressure, and melanin absorption introduces systematic skin-tone bias that pivotal datasets rarely stratify [27]. Single-lead ECG captures rhythm but not full spatial morphology, limiting infarct localization [26,34]. These are physical constraints, not software problems, so they cannot be fully resolved by better algorithms and instead require multimodal sensing, signal-quality gating, and demographically representative validation [27,31].

#### 6.1.2. False Positives, Standardization and Regulatory Fragmentation

In population-scale screening, even modest false-positive rates translate into large absolute numbers of unnecessary investigations, anxiety, and cost because positive predictive value falls as prevalence drops [9,40]. The Apple Heart Study’s 0.84 PPV and the low notification rate illustrate how screening asymptomatic low-risk populations generates many false alarms per true case [9]. This creates a downstream burden on health systems and a risk of overdiagnosis, so wearable screening is most defensible when targeted to higher-prevalence groups and paired with confirmatory testing rather than deployed indiscriminately [10,40].

#### 6.1.3. Data Privacy, Security, Ownership and Algorithmic Bias

Wearable devices generate highly sensitive data (cardiac rhythm, stress, sleep, location) transmitted across commercial cloud infrastructures with variable security [4,32]. GDPR and HIPAA provide only partial coverage of the consumer-wellness ecosystem, where much data is collected outside regulated settings, and audits document insecure transmission, unencrypted storage, and weak authentication [25,32]. Data ownership is legally unresolved, with manufacturers retaining broad rights under unread terms of service, enabling secondary commercial and insurance use without patient awareness [25,32]. Federated learning and end-to-end encryption offer mature remedies but are inconsistently implemented [32,61].

#### 6.1.4. Interoperability, Workflow Integration and Institutional Readiness

The wearable cardiovascular ecosystem comprises dozens of proprietary devices, apps, and data formats that rarely interoperate or integrate with electronic health records, so clinically relevant data are fragmented across silos and seldom reach the point of care in usable form [25,41]. The absence of universally adopted standards for data exchange, alert thresholds, and clinical workflow integration means clinicians face uncurated data streams without decision support, contributing to alert fatigue and limited uptake [25]. Emerging standards (HL7 FHIR, IEEE P11073, CDISC) and platform-level integration are addressing this, but adoption is uneven, and interoperability remains a principal barrier to translating wearable data into action [25,41,82].

#### 6.1.5. Health Equity and the Digital Divide

The benefits of wearable technology are inequitably distributed, favoring affluent, digitally connected populations while remaining inaccessible to the elderly, low-income, rural, and isolated populations who bear the greatest burden [1,53]. Devices priced at USD 200 to 800 are beyond reach for billions, and in LMICs, where 80% of cardiovascular deaths occur, cost, inconsistent smartphone ownership, unreliable internet, and limited infrastructure create near-absolute barriers; Nazir et al. found that 94% of studies were conducted in high-income countries [1,53]. Elderly adults face compounding barriers of reduced digital literacy and sensory-motor limitations against youth-optimized interfaces [3,53]. To prevent wearable and digital cardiovascular tools from widening these disparities, equity-by-design approaches, spanning inclusive interface design, digital-literacy support, and reimbursement reform, are increasingly advocated [83]. Closing this gap requires subsidized access, validation across skin tone, age, and body habits, clinician-mediated deployment, community health worker integration, and regulatory demographic-inclusion requirements [3,53,84,85]. Because equity determines whether the participatory pillar reaches those who most need it, it is a primary design consideration, not a secondary concern.

The main translational challenges, their statuses, and proposed mitigation strategies are synthesized in Table 6.

### 6.2. Future Prospects

#### 6.2.1. A Standardized Validation Protocol and Next-Generation Technology

Many of the barriers above converge on a single remedy: standardized validation. Future wearable validation studies should adopt a tiered protocol comprising a pre-specified reference standard appropriate to the claim (12-lead ECG or adjudicated patch for arrhythmia; auscultatory or invasive BP for cuffless devices); mandatory enrolment quotas ensuring representation across skin tone, age band, sex, BMI and relevant comorbidity, with pre-specified subgroup performance reporting; separation of analytical, clinical and utility endpoints, with at least one prospective external-validation cohort distinct from the development data; reporting of calibration and signal-quality coverage rather than discrimination alone; ambulatory testing conditions; and adherence to emerging reporting standards such as CONSORT-AI and SPIRIT-AI, alongside device-specific consensus criteria from EHRA, AHA, AAMI and ESH. Adoption of such a protocol would render the results comparable across devices and is a prerequisite for guideline endorsement.

In parallel, the technological trajectory points toward enhanced sensitivity, multiparameter scope, and autonomy: multiwavelength PPG with near-infrared spectroscopy for continuous hemoglobin, glucose, and lactate; piezoelectric seismocardiography for stroke volume; and energy-harvesting bioelectronics that eliminate battery constraints [30]. The AI frontier is advancing from retrospective detection toward prospective prediction via transformer models, multimodal integration of wearable biomarkers with proteomics, genomics, and imaging, and continual-learning personalization [61,87,89,90]. These directions map onto the predictive and personalized pillars that this review identifies as analytically advanced but clinically immature.

#### 6.2.2. Digital Twins, Precision Medicine, Low-Resource Deployment and Research Agenda

The most ambitious application is the digital twin, a continuously updated computational model integrating wearable data, imaging, electrophysiology, genomics, and pharmacology to enable trajectory prediction and in silico therapy testing [91]; feasibility has been shown for personalized hemodynamic forecasting, with data fidelity and validation the main remaining challenges [91]. Converging polygenic risk scores with longitudinal monitoring could flag phenotypic manifestation before clinical thresholds are crossed [61,92,93]. Global impact requires deliberate low- and middle-income-country adaptation: low-cost solar-chargeable platforms, smartphone-camera PPG, community-health-worker integration, and locally trained AI [53,85].

Critical research gaps remain substantial. Prospective randomized trials with hard cardiovascular endpoints attributable to wearable-guided management are limited, with most evidence deriving from observational or surrogate-endpoint studies [3,7,25], and output is concentrated in high-income settings and skewed toward diagnostic accuracy over outcomes [20]. Long-term adherence beyond 12 to 24 months, formal health-economic evaluation, and integration into nursing and care-coordination workflows are all under-studied [5,25,54,78]. Wearable-specific clinical guidelines specifying which patients receive which modalities and how data enter risk assessment are the most pressing translational priorities [3,25,41], and equity-centered research for underserved and LMIC populations must become a primary design priority [53,85].

**Figure 4:**
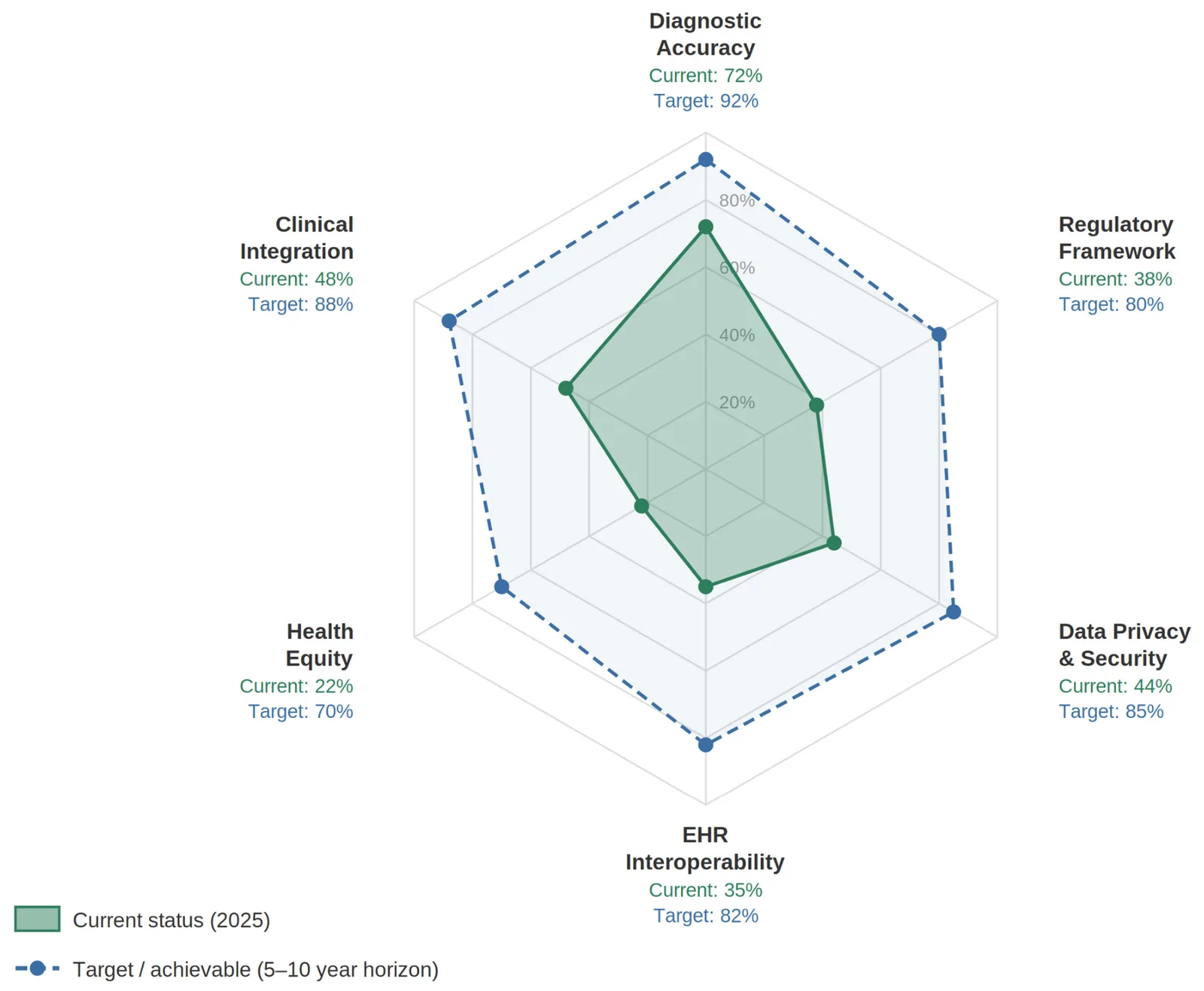
Technology maturity profile of wearable cardiovascular technology: current status and five-to-ten year target across six implementation domains.

The maturity profile shown in Figure 4 is a qualitative, author-derived synthesis rather than a quantitative measurement. Each domain was rated on a 0–100% scale by consensus of the authors, based on the validation status, evidence tier, and implementation barriers discussed in the preceding sections and summarized in Table 4 and Table 5, with the current level reflecting present-day published evidence and the target level reflecting a realistic five-to-ten-year horizon. The scores are intended to convey relative maturity across domains, not to represent an empirically measured index.

The current and projected maturity of wearable cardiovascular technology across key implementation domains is shown in Figure 4.

## 7. Conclusions

Wearable devices are reshaping cardiovascular medicine by shifting diagnosis and monitoring from episodic, clinic-centered assessment toward continuous, real-world physiological surveillance. Organized around the four pillars of predictive, preventive, personalized, and participatory care, the evidence synthesized in this review supports a single central conclusion: the four pillars are not advancing in unison, and an honest appraisal must distinguish what is already clinically proven from what remains aspirational.

Prevention and personalization rest on the firmest ground. Large-scale studies establish PPG- and ECG-based atrial fibrillation screening as a scalable, clinically validated population tool, and structured heart-failure telemonitoring is associated with meaningful reductions in hospitalization, provided it is embedded in a defined clinical-service model. Personalized monitoring, native to the continuous individual baseline that wearables uniquely provide, is clinically real in heart failure and atrial-fibrillation management, though not yet general across conditions.

The predictive pillar is analytically advanced but clinically immature. AI detection of atrial fibrillation during sinus rhythm, multiparameter decompensation forecasting, and AI-derived prognostic biomarkers such as vascular-age indices perform well internally, yet rest largely on retrospective, single-institution data and lack prospective evidence that acting on their outputs improves hard cardiovascular outcomes. The participatory pillar is supported by consistent gains in engagement, adherence, and self-management, most clearly in remote cardiac rehabilitation, but its reach is constrained by an equity gap that leaves the elderly, low-income, and low- and middle-income populations, those with the greatest cardiovascular burden, least able to benefit.

Across all four pillars, translation is limited by a shared set of barriers: accuracy variability across demographic subgroups, fragmented regulatory pathways, limited interoperability, data-privacy and security concerns, alert fatigue, algorithmic bias, and unequal access. Addressing them requires harmonized, demographically stratified validation standards, transparent regulatory frameworks, equitable implementation strategies, and clinical workflows capable of converting continuous wearable data into actionable decisions.

Ultimately, wearable cardiovascular technology is a genuine enabler of predictive, preventive, personalized, and participatory medicine, but an enabler whose promise is unevenly realized. Its future impact will depend less on further gains in sensor and algorithm performance, which are already substantial, than on rigorous prospective validation with hard endpoints, integration with telecardiology and electronic health records, deliberate attention to equity, and health-system investment in the infrastructure that turns wearable data into improved patient outcomes.

## Figures and Tables

**Table 1:** Positioning of the present review relative to representative recent reviews on wearable cardiovascular technologies, highlighting differences in focus, organizing framework, and evidence appraisal.

Review (First Author, Year, Journal)	Primary Focus	Organizing Framework	Evidence Appraisal	Key Distinction from the Present Review
Sana et al., 2020, J. Am. Coll. Cardiol. [15]	Ambulatory cardiac monitoring devices	Device and modality based	Narrative, device centric	Predates current AI maturity; no P4 mapping
Bayoumy et al., 2021, Nat. Rev. Cardiol. [16]	Smart wearables in cardiovascular care	Technology and clinical use cases	Narrative roadmap	Broad roadmap; no proven-versus-aspirational grading
Prieto-Avalos et al., 2022, Biosensors [17]	Wearables for physical heart monitoring	Sensor and signal based	Technical review	Engineering focus; limited clinical-utility appraisal
Krittanawong et al., 2019, J. Am. Coll. Cardiol. [18]	AI and deep learning in cardiovascular medicine	Algorithm taxonomy	Narrative primer	AI-centric; not wearable-specific; no P4 framing
Sîngeap et al., 2025, Life [19]	Wearable ECG for arrhythmia detection	Condition specific (AF, ST-segment)	Systematic review	Single domain; no cross-pillar synthesis
Present review, 2026, Healthcare	Wearable cardiovascular care across the P4 model	Four P4 pillars, maturity differentiated	Proven versus aspirational, per pillar, with validation tiers	Explicitly grades each pillar’s maturity; integrated-technology (pyramid) framing

**Table 2:** Operational definitions of recurring terms.

Term	Definition
Digital biomarker	Objective, quantifiable physiological or behavioral measure captured by a digital device, indicating a biological, pathological or pharmacological process. Tiered as primary (directly measured: HR, PPG/ECG waveform), secondary (computed: RMSSD, PWV, step count, SpO2 nadir) and tertiary (AI-inferred composites: PPG age, ECG age, AF-burden score, decompensation index).
Analytical validation	Demonstration that a measurement is accurate and reproducible against a reference standard (does the device measure the signal correctly?).
Clinical validation	Demonstration that the measure is associated with a meaningful health state or outcome (does the signal mean something clinically?).
Clinical-utility validation	Demonstration that using the measure improves patient outcomes beyond standard care (does acting on it help?).
ML vs. DL	Machine learning: algorithms (random forest, gradient boosting, SVM) learning from engineered or structured features. Deep learning: multilayer neural networks (CNN, RNN/LSTM, transformers) learning features directly from raw signals without manual engineering.
AI-driven prediction	Use of machine or deep learning to estimate a future state or latent condition (for example, paroxysmal-AF propensity during sinus rhythm or decompensation 7 to 14 days ahead), as distinct from detection of a present event.
AF burden	Proportion of monitored time spent in atrial fibrillation over an extended period, a continuous metric distinct from binary AF presence or absence.
Digital twin	A continuously updated computational model of an individual patient integrating wearable, imaging, electrophysiological, genomic and pharmacological data to simulate disease trajectory and test interventions in silico.
P4 medicine	A care model that is predictive (anticipates disease before symptoms), preventive (intervenes on risk continuously), personalized (tailored to individual physiology) and participatory (patient as active co-manager).

**Table 3:** Extended comparison of digital and classical cardiovascular biomarkers.

Dimension	Classical Biomarkers	Digital Biomarkers
Definition	Measurable biological indicators from blood, urine, or imaging	Objective physiological/behavioral measures from digital devices
Examples	Troponin I/T, NT-proBNP, BNP, hsCRP, LDL-C, HbA1c, eGFR	HR, HRV, AF episode burden, daily steps, SpO2 nadir, PPG age, ECG age
Data source	Laboratory analysis, conventional ECG, echocardiography	Wearable sensors, smartphone applications, digital health platforms
Measurement frequency	Episodic, at clinical encounters or acute presentations	Continuous or repeated, seconds-to-minutes intervals
Clinical context	Controlled clinical or laboratory environment	Real-world environment: home, work, exercise, sleep
Primary utility	Diagnosis, prognosis, therapeutic monitoring	Prediction, continuous surveillance, early intervention
Temporal granularity	Single time point; serial measurements days–weeks apart	Longitudinal profiling; circadian and event-triggered variation captured
Personalization	Population-derived reference ranges; limited individual baseline	Individual baseline characterization; personal deviation detection
Integration with AI	AI models applied to biomarker panels for risk prediction	AI directly derives biomarkers from raw signals and integrates with clinical data
Regulatory status	Fully validated; IVD regulated; guideline endorsed	Variable; most require further clinical validation and regulatory pathway
Key limitations	Episodic; requires clinical contact; no dynamic environmental context	Accuracy variability; standardization gaps; data privacy concerns
Clinical examples	Troponin elevation indicates ACS diagnosis; NT-proBNP indicates HF staging	HRV decline plus step-count reduction indicates early HF decompensation alert

**Table 4:** Critical comparison of regulatory frameworks: US FDA versus EU MDR 2017/745, with gap analysis.

Dimension	US FDA	EU MDR 2017/745	Key Gap/Implication
Governing framework	SaMD; 21 CFR; 510(k)/De Novo/PMA	MDR 2017/745; risk classes I to III; MDCG guidance	Different structures, leading to duplicative submissions
Consumer ECG/PPG class	Class II (De Novo) or wellness exemption	Generally Class IIa; stricter medical/wellness boundary (Rule 11)	FDA wellness exemption broader, so more devices reach market without clinical data
AI/ML software	SaMD action plan; predetermined change control for adaptive AI	Rule 11 captures most decision-support as IIa+; stringent clinical evaluation	Adaptive, continuously learning AI immaturely handled in both
Clinical evidence	510(k): substantial equivalence; De Novo: special controls	Clinical evaluation plus frequently a clinical investigation	MDR generally higher evidence bar, causing timeline and cost divergence
Review body	Centralized (FDA)	Decentralized notified bodies	MDR notified-body capacity bottleneck delays access
Post-market	MDR reporting; MAUDE	PMS plan; PSUR; EUDAMED; vigilance	More prescriptive PMS under MDR
Wellness exemption	General wellness guidance (broad)	Intended-medical-purpose test (narrower)	Same device may be exempt in US, regulated in EU
ILR classification	Class III (PMA)	Class III	Concordant (high-risk implant)
Equity/diversity mandate	Diversity action plans emerging (guidance)	No explicit demographic-validation mandate	Neither mandates subgroup validation, leaving a persistent equity gap

**Table 5:** Validation maturity and clinical readiness of wearable cardiovascular parameters and biomarkers (analytical, clinical, utility).

Parameter/Biomarker	Analytical	Clinical	Utility	Clinical Readiness	Anchor Evidence
AF detection (PPG/ECG)	Yes	Yes	Partial	Established (deployable; guideline acknowledged)	[9,10,40,42]
Step count/physical activity	Yes	Yes	Yes	Established	[14]
Resting heart rate	Yes	Yes	Partial	Established	[27]
AF burden	Yes	Partial	Partial	Emerging clinical	[29,42]
HF decompensation index (multiparameter)	Partial	Yes (programs)	Partial	Emerging	[38,39]
HRV metrics	Partial	Partial	No	Emerging (standardization gaps)	[27,32]
AI-ECG LV dysfunction screening	Yes	Partial	No	Emerging screening	[13]
Wearable SpO2 (<90%)	Partial	Partial	No	Adjunctive	[31]
Cuffless blood pressure	Partial	No	No	Investigational	[31]
Thoracic bioimpedance	Partial	Early	No	Investigational	[39]
PPG age/ECG age	Partial	Observational assoc.	No	Research	[36,43]

**Table 6:** Challenges in wearable cardiovascular technology: status and proposed solutions.

Challenge Category	Description	Status	Proposed Solution	Key References
Signal accuracy, motion artefact	PPG signal contamination during movement; ECG noise from electrode contact variability	Partially resolved; residual error at high intensity	Accelerometer-based artifact subtraction; deep learning signal separation; multi-site sensor fusion	[19,27,31]
Population performance variability	Reduced accuracy in darker skin tones, elderly, obese, and arrhythmic patients	Unresolved; most validation studies in homogeneous populations	Mandatory demographic-stratified validation; melanin-adaptive PPG wavelengths; population-specific AI retraining	[3,19,27,86]
False-positive alert burden	High notification rates generating unnecessary downstream investigation and patient anxiety	Partially resolved through higher PPV thresholds; clinical burden persists	AI-powered pre-screening triage; Bayesian clinical context integration; two-step confirmation protocols	[3,9,41]
Regulatory fragmentation	Divergent FDA, EU MDR, and national frameworks for device classification and approval	In progress, IMDRF SaMD harmonization underway	International regulatory convergence; harmonized evidence standards for wearable ECG and BP devices	[3,25]
Validation protocol heterogeneity	No universally adopted condition-specific validation protocols; inter-study accuracy ranges 71–99%	In progress, EHRA, AHA, IEEE initiatives underway	Condition-specific international validation standards; mandatory head-to-head device comparison studies	[3,19,25,31]
Data privacy and cybersecurity	Sensitive cardiovascular data transmitted via insecure Bluetooth and stored in commercial cloud platforms	Partially addressed by GDPR/HIPAA; consumer wellness devices largely unregulated	End-to-end encryption mandate; health data classification extension to consumer wearables; federated learning	[25,32]
Data ownership and consent	Manufacturers retain broad secondary use rights; patients unaware of commercial data utilization	Unresolved; no standardized data governance framework internationally	Explicit consent frameworks; patient data rights; standardized wearable data ownership policies	[25,32]
Algorithmic bias	AI training datasets over-representing white, male, young, high-income populations	Partially acknowledged; few corrective mandates exist	Mandatory training data diversity standards; fairness-constrained model optimization; demographic performance reporting	[3,84,86]
Interoperability, data standards	Proprietary device data formats incompatible with EHR systems and with each other	In progress, FHIR adoption expanding but inconsistent	Universal FHIR DeviceMetric adoption mandate; standardized physiological data ontologies; open API requirements	[25,27,32]
EHR integration and alert fatigue	Clinical data volumes overwhelming traditional EHR display; clinician alert desensitization	Partially resolved for HF telemonitoring; unresolved for broader applications	AI-mediated data summarization; alert triage algorithms; dedicated program coordinators; EHR wearable dashboard standards	[25,41,87]
Staff training and institutional readiness	Clinicians and nurses lack wearable data interpretation training and data governance literacy	Early stages; no standardized training curricula exist	Wearable cardiology competency frameworks; institutional readiness assessment tools; reimbursement for monitoring coordination	[25,41,88]
Health equity, access barriers	Device costs and digital literacy requirements exclude elderly, low-income, and LMIC populations	Unresolved; 94% of studies conducted in high-income countries	Subsidized device access; clinician-mediated deployment; community health worker integration; LMIC-adapted low-cost devices	[1,53,85]
Evidence gaps, hard endpoints	Most evidence based on observational cohorts and surrogate endpoints; few RCTs with CV mortality data	In progress, prospective trials underway	Large multicenter RCTs with hard cardiovascular endpoints; long-term adherence and cost-effectiveness studies	[3,7,25]

## Data Availability

No new data were created or analyzed in this study. Data sharing is not applicable to this article.

## Supplementary Materials

The following supporting information can be downloaded at https://www.mdpi.com/article/10.3390/healthcare14182894/s1: Table S1: Cardiovascular monitoring devices, one representative device per row, with monitored parameters, reported accuracy, regulatory status and supporting citation; Table S2: Key clinical studies on wearable devices in cardiovascular medicine: study design and characteristics; Table S3: Key clinical studies on wearable devices in cardiovascular medicine: primary outcomes, diagnostic accuracy, and level of evidence; Table S4: AI algorithms applied to wearable cardiovascular data: device or signal source, key extracted features and preprocessing; Table S5: AI algorithms applied to wearable cardiovascular data: diagnostic accuracy and validation status; Table S6: Comparative analysis of clinical applications: atrial fibrillation and acute coronary syndrome; Table S7: Comparative analysis of clinical applications: heart failure, hypertension, cardiac rehabilitation and ventricular-arrhythmia monitoring; Table S8: Additional studies contributing to the evidence base: AI and digital health applications in cardiovascular medicine.

## Abbreviations

The following abbreviations are used in this manuscript:

AAMI	Association for the Advancement of Medical Instrumentation
ACS	acute coronary syndrome
AF	atrial fibrillation
AHA	American Heart Association
AI	artificial intelligence
AI-ACS	artificial intelligence-based acute coronary syndrome detection
AI-ECG	artificial intelligence-enabled electrocardiography
AVNRT	atrioventricular nodal re-entrant tachycardia
AVRT	atrioventricular re-entrant tachycardia
BNP	B-type natriuretic peptide
BP	blood pressure
CAD	coronary artery disease
CDISC	Clinical Data Interchange Standards Consortium
CE	Conformité Européenne
CHA2DS2-VASc	Congestive heart failure, Hypertension, Age ≥75, Diabetes, Stroke/TIA, Vascular disease, Age 65–74, Sex category
CI	confidence interval
CKD	chronic kidney disease
CNN	convolutional neural network
CPAP	continuous positive airway pressure
CR	cardiac rehabilitation
CVD	cardiovascular disease
DNN	deep neural network
ECG	electrocardiogram
EDA	electrodermal activity
EF	ejection fraction
EHR	electronic health record
EHRA	European Heart Rhythm Association
EMG	electromyography
EU	European Union
FDA	Food and Drug Administration
FHIR	Fast Healthcare Interoperability Resources
GDPR	General Data Protection Regulation
GUCH	grown-up congenital heart disease
HF	heart failure
HIPAA	Health Insurance Portability and Accountability Act
HR	heart rate
HRV	heart rate variability
HbA1c	glycated hemoglobin
hsCRP	high-sensitivity C-reactive protein
IEEE	Institute of Electrical and Electronics Engineers
ILR	implantable loop recorder
IMDRF	International Medical Device Regulators Forum
IVD	in vitro diagnostic
LBBB	left bundle branch block
LDL-C	low-density lipoprotein cholesterol
LF/HF	low-frequency/high-frequency ratio
LMIC	low- and middle-income country
LOE	level of evidence
LSTM	long short-term memory
LV	left ventricular
MAE	mean absolute error
MCOT	mobile cardiac outpatient telemetry
MDR	Medical Device Regulation
MI	myocardial infarction
ML	machine learning
NT-proBNP	N-terminal pro-B-type natriuretic peptide
OSA	obstructive sleep apnea
P4 medicine	predictive, preventive, personalized, and participatory medicine
PCI	percutaneous coronary intervention
PHQ-9	Patient Health Questionnaire-9
PMA	premarket approval
PPG	photoplethysmography
PPV	positive predictive value
PTT	pulse transit time
QT interval	ventricular depolarization–repolarization interval
RCT	randomized controlled trial
RMSSD	root mean square of successive differences
RNN	recurrent neural network
ROC	receiver operating characteristic
SaMD	software as a medical device
SCD	sudden cardiac death
SDNN	standard deviation of normal-to-normal intervals
SF-36	36-Item Short Form Health Survey
SHAP	SHapley Additive exPlanations
SpO2	peripheral oxygen saturation
ST segment	segment between ventricular depolarization and repolarization
STEMI	ST-elevation myocardial infarction
SVT	supraventricular tachycardia
VT	ventricular tachycardia
XAI	explainable artificial intelligence
