# Supplementary material for: Wearable Devices in Cardiovascular Care: A Narrative Review of the Transition Toward Predictive, Preventive, Personalized, and Participatory Medicine
Source: Healthcare (Basel). 2026 Sep 8;14(18):2894. doi: 10.3390/healthcare14182894 (PMC13606132; doi:10.3390/healthcare14182894)
Supplement: Supplementary file 1 [file healthcare-14-02894-s001.zip › healthcare-4468268-supplementary.pdf]

Supplementary Materials

Wearable Devices in Cardiovascular Care: A Narrative Review of the Transition Toward Predictive, Preventive, Personalized, and Participatory Medicine

Simona Steliana Tudor [1], Ancuta Elena Tupu [1,*], Alice Elena Munteanu [2,3,*], Claudia Simona Stefan [1] and Ionela Daniela Ferțu [1]

[1] Faculty of Medicine and Pharmacy, Medical-Pharmaceutical Research Center, "Dunarea de Jos" University of Galati, 800008, Galati, Romania; steliana.tudor@ugal.ro (S.S.T.); claudia.stefan@ugal.ro (C.S.S.); ionela.fertu@ugal.ro (I.D.F.)
[2] Faculty of Medicine, Titu Maiorescu University, 031593, Bucharest, Romania
[3] Central Military Emergency University Hospital „Dr. Carol Davila", 010825, Bucharest, Romania
* Correspondence: anca.tupu@ugal.ro (A.E.T.); dralicepopescu@yahoo.com (A.E.M.)

Table S1. Cardiovascular monitoring devices, one representative device per row, with monitored parameters, reported accuracy, regulatory status and supporting citation.

Tier	Representative device	Monitored parameters	Reported accuracy	Regulatory status	Ref.
Consumer / wellness	Apple Watch (S9/Ultra 2)	HR, HRV, single-lead ECG, PPG, SpO2, activity	HR MAE 2–5 bpm; AF irregular-pulse PPV 0.84	FDA De Novo (ECG/AF); CE wellness	[3,9]
Consumer / wellness	Fitbit Sense 2	HR, HRV, PPG, SpO2, EDA, activity	AF PPV 98.2% (with patch confirmation)	FDA-cleared (irregular rhythm); CE wellness	[10]
Consumer / wellness	Samsung Galaxy Watch 6	HR, single-lead ECG, PPG, cuffless BP, SpO2	Manufacturer data, independent validation pending	FDA De Novo (ECG); CE	[3]
Consumer / wellness	Garmin Fenix 7	HR, HRV, PPG, SpO2, activity	HR accurate at rest; degrades during exercise and AF	CE wellness	[3,27]
Medical-grade	Zio XT patch (iRhythm)	Continuous single-lead ECG (up to 14 d), arrhythmia	Detects arrhythmia in 34–71% of Holter-negative cases	FDA 510(k); CE Class IIa	[5,26]
Medical-grade	KardiaMobile 6L	6-lead intermittent ECG, AF detection	Sens. 71–99%, spec. 73–99% for AF (wearable ECG device class)	FDA-cleared; CE	[19]
Medical-grade	Philips/BioTel MCOT	Continuous mobile cardiac telemetry	Holter-equivalent arrhythmia classification	FDA-cleared; CE Class IIa	[26]
Implantable (reference comparator)	Medtronic Reveal LINQ (ILR)	Continuous single-lead ECG up to 3 y, AF burden	AF detection 3–4x Holter; CRYSTAL AF 30% vs 3% at 3 y	FDA PMA; CE Class III	[29]
Implantable (reference comparator)	Abbott Confirm Rx (ILR)	Continuous ECG, smartphone-paired	High-fidelity long-term arrhythmia detection	FDA PMA; CE Class III	[7,26]

Tier	Representative device	Monitored parameters	Reported accuracy	Regulatory status	Ref.
Textile / soft	Hexoskin smart garment	1-lead ECG, respiratory rate, HR, activity	ECG comparable to Holter in controlled settings	CE wellness/medical (variant)	[30]
Chest strap	Polar H10	HR, HRV, respiratory rate	HR MAE <1 bpm vs ECG reference	CE wellness / FDA exempt	[17]
Soft bioelectronic	MC10 BioStamp / Sibel	ECG, EMG, EDA, temperature, optical PPG	Research-grade; comparative clinical data limited	Investigational / FDA Breakthrough	[30]
Cuffless BP	Omron HeartGuide	Oscillometric wrist BP, HR	Mean diff. 0.3–7.8 mmHg systolic, 0.2–8.6 mmHg diastolic vs cuff	CE Class IIa (limited markets)	[31]
Cuffless BP	Huawei Watch D	Cuff-inflation wrist BP, HR	Manufacturer data; independent validation pending	CE (limited markets)	[3,31]

Table S2. Key clinical studies on wearable devices in cardiovascular medicine: study design and characteristics.

Author, Year	Design	N	Device	Parameter	Application
Perez et al., 2019 [9]	Prospective cohort	419,297	Apple Watch	PPG	AF screening
Lubitz et al., 2022 [10]	Prospective cohort	455,699	Fitbit	PPG	AF screening
Tison et al., 2018 [44]	Retrospective + prospective	9,750	Smartwatch	PPG	Passive AF detection
Guo et al., 2019 [40]	RCT	3,207	Mobile PPG app	PPG	AF screening (primary care)
Attia et al., 2019 [12]	Retrospective	180,922	ECG (AI)	12-lead ECG	AF detection in sinus rhythm
Attia et al., 2019 [13]	Retrospective	97,829	ECG (AI)	12-lead ECG	LV dysfunction screening
Galloway et al., 2019 [65]	Retrospective	61,965	ECG (AI)	2-lead ECG	Hyperkalaemia screening
Choi et al., 2024 [34]	Prospective	71	Smartwatch (9-lead)	ECG + AI	ACS detection
Hannun et al., 2019 [11]	Retrospective	53,549 ECGs	Ambulatory ECG monitor	1-lead ECG (DNN)	12-class arrhythmia classification
Devin et al., 2025 [38]	Systematic review	Variable	Multiparameter wearables	HR, activity, SpO2, weight	HF decompensation prediction
Sîngeap et al., 2025 [19]	Systematic review	Variable	Multiple wearable ECG	ECG	AF and ST-segment detection
Nie et al., 2025 [36]	Prospective cohort	>50,000	Wearable PPG	PPG age (AI)	Cardiovascular mortality prediction

Author, Year	Design	N	Device	Parameter	Application
Pizarro et al., 2025 [39]	Prospective cohort	Variable	Remote monitoring	Multi-parameter	HF remote management

Table S3. Key clinical studies on wearable devices in cardiovascular medicine: primary outcomes, diagnostic accuracy, and level of evidence.

Author, Year	Primary outcome	AUC / Sens. / Spec.	Key limitation	LOE
Perez et al., 2019 [9]	PPV 0.84 for irregular pulse notification	Sens. 71% / Spec. 99%	Mostly white, young, male cohort	High
Lubitz et al., 2022 [10]	PPV 98.2% (wearable + patch)	Sens. 98% / Spec. 90%	No independent ECG confirmation arm	High
Tison et al., 2018 [44]	Sensitivity 98.5%	AUC 0.97	Single-centre validation	Moderate
Guo et al., 2019 [40]	4.6x AF detection increase vs. usual care	Sens. 97.4% / Spec. 97.4%	China only; selection bias possible	High
Attia et al., 2019 [12]	AUC 0.87–0.90	Sens. 79–82% / Spec. 79–83%	Single institution; generalisability unclear	High
Attia et al., 2019 [13]	AUC 0.93 for EF ≤35%	Sens. 86.3% / Spec. 85.7%	Retrospective; single institution	High
Galloway et al., 2019 [65]	AUC 0.853–0.883	Sens. 90% / Spec. 63%	CKD population only	Moderate
Choi et al., 2024 [34]	AUC 0.987 vs. 0.991 (12-lead, p = 0.745)	AUC 0.987	Very small sample; single centre	Moderate
Hannun et al., 2019 [11]	Average AUC 0.97; surpasses cardiologists	Sens. 85% / Spec. 97%	Limited demographic diversity	High
Devin et al., 2025 [38]	AUC 0.78–0.92; 7–14-day lead time	Pooled AUC 0.85	Study heterogeneity; variable AI models	Moderate
Sîngeap et al., 2025 [19]	Sensitivity range 71–99% for AF	Spec. 73–99%	High inter-study heterogeneity	Moderate
Nie et al., 2025 [36]	PPG age discordance predicts CV mortality	HR 1.42 per 5-year PPG age excess	Indirect endpoint; observational	Moderate
Pizarro et al., 2025 [39]	11–32% HF hospitalisation reduction	—	Heterogeneous interventions	Moderate

Table S4. AI algorithms applied to wearable cardiovascular data: device or signal source, key extracted features and preprocessing.

Model / algorithm	Application	Device / signal source	Key extracted features	Preprocessing / conditioning	Ref.
CNN (34-layer ResNet)	12-class arrhythmia classification	Ambulatory 1-lead ECG patch	Learned beat and rhythm morphology (end-to-end)	Bandpass filtering, resampling, beat segmentation	[11]
CNN	AF detection in sinus rhythm	12-lead ECG (clinic)	Subtle P-wave and repolarization substrate features	Lead normalization, baseline-wander removal	[12]

Model / algorithm	Application	Device / signal source	Key extracted features	Preprocessing / conditioning	Ref.
CNN	LV systolic dysfunction screening	12-lead ECG	Latent depolarization and repolarization features	Standard ECG denoising, normalization	[13]
CNN	Hyperkalaemia detection	2-lead ECG	T-wave morphology features	Filtering, segmentation	[65]
Deep learning (image-based)	ACS / STEMI detection	9-lead smartwatch ECG (reconstructed)	ST-segment, reconstructed-lead morphology	Lead reconstruction, image transformation	[34]
CNN + PPG	Passive AF detection	Consumer smartwatch PPG	RR-interval irregularity, pulse morphology	Motion-artifact removal, signal-quality gating	[44]
LSTM recurrent network	HF decompensation prediction	Multiparameter wearable + scale	HR, activity, SpO2, weight temporal trends	Resampling, normalization, trend extraction	[38]
CNN + PPG morphology	PPG age (vascular ageing)	Wearable PPG waveform	Waveform shape and ageing features	Beat alignment, normalization, denoising	[36]
CNN	Aortic stenosis detection	12-lead ECG	Learned valvular substrate features	Standard ECG denoising, normalization	[66]
Deep learning (multimodal)	Sudden cardiac arrest risk	Wearable ECG + clinical data	Fused ECG morphology and clinical risk features	Signal-quality control, feature harmonization	[57]
RF + gradient boosting	10-year multimodal CVD risk	Wearable + biomarker + imaging	Engineered multimodal features	Feature scaling, missing-data handling	[80]

Table S5. AI algorithms applied to wearable cardiovascular data: diagnostic accuracy and validation status. Rows are listed in the same order as in Table S4.

Model / algorithm	AUC	Sensitivity	Specificity	Dataset size	External validation	Ref.
CNN (34-layer ResNet)	0.97	85%	97%	53,549 ECGs	Partial	[11]
CNN	0.87–0.90	79–82%	79–83%	649,931 ECGs (180,922 patients)	Multi-site Mayo	[12]
CNN	0.93	86.3%	85.7%	97,829 ECGs	Multi-site Mayo	[13]
CNN	0.853–0.883	90%	63%	1.58M ECGs (61,965 CKD validation)	3 Mayo sites	[65]
Deep learning (image-based)	0.987	94%	96%	71 patients	None	[34]
CNN + PPG	0.97	98.5%	90.2%	9,750 participants	Prospective arm	[44]

Model / algorithm	AUC	Sensitivity	Specificity	Dataset size	External validation	Ref.
LSTM recurrent network	0.78–0.92	Variable	Variable	Pooled (systematic review)	Limited	[38]
CNN + PPG morphology	—	—	—	>50,000 participants	2 cohorts	[36]
CNN	0.85	77%	81%	38,676 ECGs	External	[66]
Deep learning (multimodal)	0.82–0.88	74%	85%	Variable	Limited	[57]
Random forest + gradient boosting	C-stat 0.84–0.91	—	—	>100,000	Multi-cohort	[80]

Table S6. Comparative analysis of clinical applications: atrial fibrillation and acute coronary syndrome.

Application	Device / modality	Monitored signals	AI algorithm & features	Representative performance	Validation tier / LOE	Key finding & limitation
AF: population screening	Consumer smartwatch	PPG (± single-lead ECG)	CNN / irregularity; RR-interval, pulse morphology	Sens. 93–98%; spec. 88–98%; PPV 0.84–0.98	Analytical + clinical + partial utility; LOE 2	Scalable and validated; PPV prevalence-dependent; ectopy false positives; non-representative cohorts
AF: detection in sinus rhythm	Clinic / wearable ECG	12- / single-lead ECG	CNN; subtle P-wave and repolarization substrate	AUC 0.87–0.90; sens/spec 79–83%	Analytical; LOE 3	Pre-emptive AF identification; single-institution, external validity untested
AF: burden quantification	ECG patch / ILR comparator	Continuous ECG	Rhythm classification and time-in-AF	Burden >5.5 h/day linked to higher stroke risk	Clinical; LOE 2–3	Informs anticoagulation (EHRA); ILR not wearable
ACS: triage	Smartwatch 9-lead (reconstructed)	Reconstructed-lead ECG	Deep learning; ST-segment morphology	AUC 0.987 vs 0.991 for 12-lead (p = 0.745)	Analytical; LOE 3	Equivalent in a tiny cohort (n = 71); cannot localize infarct; lower sensitivity for posterior or LBBB-masked MI; not approved for ACS diagnosis

References by row: AF, population screening [3,9,10,40,44]; AF, detection in sinus rhythm [12]; AF, burden quantification [29,42]; ACS, triage [19,28,34].

Table S7. Comparative analysis of clinical applications: heart failure, hypertension, cardiac rehabilitation and ventricular-arrhythmia monitoring.

Application	Device / modality	Monitored signals	AI algorithm & features	Representative performance	Validation tier / LOE	Key finding & limitation
HF: decompensation prediction	Multiparameter wearable + scale	HR, activity, SpO2, weight, (bioimpedance)	LSTM / ML fusion; trend features	AUC 0.78–0.92; 7–14-day lead time	Clinical; LOE 2–3	Multiparameter much better than single-parameter; effect partly organizational (service model)
HF: programme outcomes	Remote monitoring suite	Multiparameter	Tiered alert (rule + ML)	11–32% hospitalization reduction	Clinical + utility; LOE 1–2	Benefit tied to structured oversight; heterogeneous interventions
Hypertension: cuffless BP	Wrist cuffless / PTT	PPG, PTT, (ECG)	ML regression; pulse-transit features	Mean diff. 0.3–8.6 mmHg vs cuff	Analytical only; LOE 3–4	Fails AAMI/ESH thresholds; adjunctive trend use only; unvalidated in pregnancy, elderly, AF
Cardiac rehabilitation	Chest strap / patch / smartwatch	HR, HRV, accelerometry, ECG	Rule-based + ML; HR zones, duration	Equivalent peak VO2 vs facility CR; 72–85% completion	Clinical; LOE 1–2 (efficacy)	Home CR non-inferior; supervision and safety monitoring needed
Ventricular arrhythmia / SCD	ECG patch	Continuous ECG	CNN morphology; QRS/QT features	Early evidence; no pooled metrics	Analytical / exploratory; LOE 4	Promising for ectopy, NSVT, QT; immature for SCD risk decisions

References by row: HF, decompensation prediction [38]; HF, programme outcomes [39,41,49]; hypertension, cuffless BP [3,31]; cardiac rehabilitation [54,55]; ventricular arrhythmia / SCD [26,57,58].

Table S8. Additional studies contributing to the evidence base: AI and digital health applications in cardiovascular medicine.

Author, Year	Application domain	Key contribution to this review
Baig et al., 2017 [82]	Wearable patient monitoring systems	Systematic review identifying core implementation challenges (interoperability, alert management, staff training)
Krittanawong et al., 2019 [18]	Deep learning in cardiovascular medicine	Practical primer establishing the CNN/RNN taxonomy and clinical deployment framework
Oikonomou & Khera, 2024 [81]	AI-enhanced patient evaluation	Frames the art-science convergence in AI cardiovascular diagnostics; supports the explainability discussion
Biswas et al., 2025 [21]	AI for cardiovascular care	Documents real-world AI implementation across cardiology subspecialties; supports the clinical-implications discussion
Patel et al., 2025 [76]	AI in cardiology, systematic review	Ethical considerations and implementation challenges; supports the regulatory and algorithmic-bias discussion
Shuja et al., 2024 [64]	AI advancements in cardiology	Synthesis of AI diagnostic, treatment, and patient-care applications

Author, Year	Application domain	Key contribution to this review
Dubatovka & Buhmann, 2022 [45]	AF detection from single-lead ECG	Deep learning of the cardiac cycle for AF classification; extends single-lead ECG evidence
Țica et al., 2025 [46]	AI in AF diagnosis and management	AI diagnostic and management framework for AF
Khaja et al., 2025 [47]	AI in AF diagnostics, systematic review	Meta-level evidence for AI-powered AF diagnostics
Canino et al., 2025 [58]	AI in cardiac electrophysiology	Engineering-clinical interface for AI in electrophysiology
Yoon et al., 2023 [50]	AI in heart failure	Historical, current, and future AI applications in HF
Idris-Agbabiaka et al., 2025 [51]	AI-assisted heart failure management	Case studies and clinical AI implementation in HF
Wan et al., 2026 [55]	AI in digital health for cardiac rehabilitation	AI-optimized remote CR framework
Velandia et al., 2025 [68]	AI and ECG for CVD diagnosis, systematic review	Systematic evidence for ECG-based AI across cardiovascular conditions
Neupane et al., 2025 [69]	AI in ECG interpretation, outpatient scoping review	Outpatient-setting AI-ECG evidence
Kwon et al., 2020 [66]	AI-ECG for aortic stenosis detection	AUC 0.85 for aortic stenosis from 12-lead ECG
Cho et al., 2020 [67]	AI for MI detection from 6-lead ECG	Reduced-lead AI-ECG for STEMI
Kim et al., 2025 [70]	AI-ECG for perioperative myocardial ischemia	Scoping review of perioperative AI-ECG
Bozzi et al., 2026 [43]	AI ECG-age as biomarker of mortality, meta-analysis	Systematic evidence for ECG biological age as a CV mortality predictor
Xu et al., 2026 [93]	Precision cardiovascular medicine with big data and AI	Big data-AI convergence framework
Hampe et al., 2019 [71]	ML for coronary artery disease in cardiac CT	Survey of ML in CT-based CAD assessment
Motwani et al., 2016 [72]	ML for all-cause mortality prediction	Landmark multicenter ML mortality registry study
Ouyang et al., 2020 [73]	Video-based AI for cardiac function assessment	Beat-to-beat EF assessment via AI video
Van Velzen et al., 2020 [74]	Deep learning for calcium scoring in CT	Multi-protocol AI calcium scoring validation
Zhang et al., 2018 [75]	Fully automated echocardiogram interpretation	First large-scale AI echocardiography validation

Abbreviations: ACS, acute coronary syndrome; AF, atrial fibrillation; AUC, area under the receiver operating characteristic curve; BP, blood pressure; CKD, chronic kidney disease; CNN, convolutional neural network; CR, cardiac rehabilitation; CVD, cardiovascular disease; ECG, electrocardiogram; EDA, electrodermal activity; EF, ejection fraction; EHRA, European Heart Rhythm Association; EMG, electromyography; HF, heart failure; HR, heart rate (hazard ratio where stated); HRV, heart rate variability; ILR, implantable loop recorder; LBBB, left bundle branch block; LOE, level of evidence; LSTM, long short-term memory network; LV, left ventricular; MAE, mean absolute error; MCOT, mobile cardiac outpatient telemetry; MI, myocardial infarction; NSVT, non-sustained ventricular tachycardia; PPG, photoplethysmography; PPV, positive predictive value; PTT, pulse transit time; RCT, randomised controlled trial; RF,

random forest; SCD, sudden cardiac death; Sens., sensitivity; Spec., specificity; SpO_2, peripheral oxygen saturation; STEMI, ST-elevation myocardial infarction; VO_2, oxygen uptake.
